# Supplementary material for: Lay perspectives of the open-label placebo rationale: a qualitative study of participants in an experimental trial
Source: BMJ Open. 2021 Aug 17;11(8):e053346. doi: 10.1136/bmjopen-2021-053346 (PMC8375733; doi:10.1136/bmjopen-2021-053346)
Supplement: Supplementary data [file bmjopen-2021-053346supp001.pdf]

eAppendix 1. Provided rationales

Translated from German into English

Deceptive Placebo (DP) group:

Now, before you take the pain measurements for the second time, I would like to explain you the treatment approach. You will now receive a cream for pain relief. It is a generic product with the active ingredient lidocaine, which is also contained in creams like “Stilex”. The “antidolor cream” is used to prevent and treat painful and itchy skin conditions such as first-degree burns, minor non-extensive sunburn and insect bites. The effect of lidocaine has been proven in multiple high-quality studies.

OPR- group:

Now, before you take the pain measurements for the second time, you will receive a placebo cream. This means that you will be given a cream that does not contain any medicinal active ingredient and that is therefore inactive.

OPR+ group:

Now, before we take the pain measurements for the second time, I would like to explain you the treatment approach. You will now receive a placebo cream. Have you ever heard of placebo? Placebo is something that does not contain an active ingredient and is inactive, but still works. Now I will give you an overview of newest research findings:

First discussion point:

It has been shown that placebos can work just as well as real drugs, especially in the fields of pain, Parkinson's disease, depression, migraine and asthma. For example, in the field of pain: Research indicates that the placebo effect is involved in 50% of the effect of pain pills and creams [Mention two studies: Colloca et al., 2013; Benedetti et al., 2001].

Conclusively, placebos lead to measurable biological reactions in the body and are much more than just imagination. So, you could say that placebos boost the "body's own pharmacy".

But it gets even more interesting - placebos even work when you know it's a placebo. This is the approach we are taking in our experiment. For our study, we are working closely with the research group from Harvard. In a landmark study of 2010, it was shown that patients with

irritable bowel syndrome had a significant decrease in symptoms, even when they were openly informed that they were receiving a placebo [Kaptchuk et al., 2010].

Second discussion point:

Now, I would like to tell you about *how* placebos work. One explanation for these processes is classical conditioning. Maybe you heard about the experiment of Pavlov and his dog. The dog is given food several times with a bell ringing. Later, the pure bell ringing leads to salivation, even if the dog does not receive food. Comparable is the physiological reaction of our body to placebos: From an early age we learn that the application of a cream and the effect are associated. Finally, the mere application of the cream results in pain relief. Similar to the Pavlov's dog, we therefore perform a ritual.

Third discussion point:

The discussed treatment approach may sound a bit unusual at first sight. Another plus point of placebos is that a positive attitude helps but is not necessary.

eAppendix 2. Topic guide of the qualitative interview

1. Discourse

In general:

In your own words: what do you imagine a placebo to be?	
How do you think placebos work?	
What could be the cause/reason?	

Related to the experiment:

How did the experiment influence your understanding of placebos?	

2. Project

In general:

To what extent do placebos occur in your everyday life? (Active use of the placebo effect - e.g., in the form of home remedies)	
How significant are these effects and implications of a placebo for you?	

Related to the experiment:

Now if you had pain (e.g., heat pain, burning sensation) - would you use a cream described as "placebo"?	
If yes: Would you have taken this cream before experiencing this experiment?	

3. Selfhood

In general:

Do you think you respond particularly well/badly to placebos? Why?	
How much control do you think you have over the effect of a placebo?	

Related to the experiment:

What do you think - did the placebo cream have an effect on pain perception in this study? If so, what was the effect?	

4. Embodiment

In general:

Do you believe that a placebo can have an "effective" physical effect or is it merely an "imaginary" effect, i.e., a deception (of the senses)?	

Related to the experiment:

What was it like for you? Please describe the physical sensations of using the placebo cream.	

5. Sociality

In general:

To which people in your social environment would you recommend placebos? Why?	

Related to the experiment:

Will you tell people at home (with friends or relatives) about your experience with placebos that you had in the experiment?	
How will this make you feel towards the other person?	

6. Spatiality

In general:

Can you describe to me an environment or place where you feel, a placebo would work particularly well?	

Related to the experiment:

What impact did it have on you that you received the placebo in the context of an experiment and not in the "real world"?	

7. Temporality

In general:

How long do you think placebos last?	
When you think back - what significant memories with placebos come to mind? (e.g., comforting songs in childhood, remedies for a cold)	

Related to the experiment:

A glimpse into the future: Do you take anything away from the experiment and from this conversation with respect to placebos?	

eTable 1. Superordinate categories, main categories and categories

Superordinate categories, main categories and categories

Superordinate Categories	Main Categories	Categories	Frequencies		
			OPR-	OPR+	DP
1. The placebo concept.	1.1. Descriptive	Deception	4	6	10
		Study design component	1	0	1
		Inertness	7	9	6
		Substitute	2	0	0
	1.2. Effectiveness	Regularity of intake is necessary for effectiveness	2	1	2
		Sham effect	0	0	2
		Effect on body	9	3	7
		Psychological effect	3	1	1
		Paradox: Effect without active ingredient	6	5	1
		Effect-free	5	0	1
	1.3 Specific examples	Autosuggestion	1	1	1
		Spirituality	1	1	1
		Drug	0	4	2
		Interpersonal component	2	2	5
		Esotericism	1	0	1
		Deception	0	0	1
		Advertisement	0	0	1
		Complementary medicine	8	8	9
		Action	0	4	6
		Activity	2	3	6
		Food supplements	0	2	2
		Mindfulness	1	0	0
		Food products	9	8	11
2. Open-label placebo rationale.	2.1 First discussion point: “Placebos are powerful”	Less efficacy due to lack of deception	4	2	5
		OLP administration mitigates expectancy/belief	4	2	5
		OLPs work	0	1	0
		Surprise about OLPs effectiveness	0	2	0
		Effect of deceptive placebos lasts longer in contrast to OLPs	0	2	0
		Effect due to deceptive placebos, less effect due to OLPs	0	2	0
	2.2 Second discussion point: “The body automatically responds to placebos like Pavlov’s dog”	Relationship	9	6	7
		Imagination	0	2	2
		Psychological factors	11	11	7
		Misattribution	1	0	0
		Mind-body-interaction	22	17	13
		Conditioning	4	11	2
		Ritual/action	3	5	1
		Openness/personality/attitudes	37	24	26
		Expectancy/belief/hope	28	27	30
	2.3 Third discussion	Depending on the experience	2	6	1
		If it helps and does no harm	0	0	1

	point: "A positive attitude helps but is not necessary"	If it's the only option	2	3	2
		It's worth a try	3	2	0
		Worth to see what happens	0	2	0
		As long as one believes that it helps	2	0	3
		Depending on the person	2	0	1
		Depending on the situation	0	1	0
		Depending on symptoms / disorder	4	4	3
3. Experiences of taking part in an open-label placebo trial.	3.1. Reaction to (open-label) placebo	When administered openly, skepticism arises as to whether an ingredient is not in the pill after all	2	1	0
		OLPs do not elicit wellbeing	0	1	0
		OLPs as something novel	1	6	0
		OLPs are incomprehensible/strange/crazy	5	1	1
		Openness towards OLPs	0	1	0
		Open administration is less popular	0	1	0
		OLP rationale is essential	0	3	0
		Placebo as something unknown	0	0	2
	3.2. Efficacy and effectiveness during the experiment	No efficacy due to the lack of deception	0	1	0
		Efficacy unlikely	1	3	0
		Efficacy only assumed posthoc	0	0	2
		Efficacy possible	4	1	4
		Efficacy unclear	1	3	0
		No objective efficacy (measures), but subjective efficacy (own sensation)	0	0	1
		Efficacy likely	1	0	4
		Positive experience due to cooling/experimental factors	4	3	4
		Positive experience on a psychological level	1	2	0
		No experienced efficacy in the experiment	9	9	5

Note. DP = deceptive placebo; OPR- = open-label placebo without rationale; OPR+ = open-label placebo with rationale.
