# Supplementary material for: Lay perspectives of the open-label placebo rationale: a qualitative study of participants in an experimental trial
Source: BMJ Open. 2021 Aug 17;11(8):e053346. doi: 10.1136/bmjopen-2021-053346 (PMC8375733; doi:10.1136/bmjopen-2021-053346)
Supplement: Author's manuscript [file bmjopen-2021-053346.draft_revisions.pdf]

BMJ Open is committed to open peer review. As part of this commitment we make the peer review history of every article we publish publicly available.

When an article is published we post the peer reviewers' comments and the authors' responses online. We also post the versions of the paper that were used during peer review. These are the versions that the peer review comments apply to.

The versions of the paper that follow are the versions that were submitted during the peer review process. They are not the versions of record or the final published versions. They should not be cited or distributed as the published version of this manuscript.

BMJ Open is an open access journal and the full, final, typeset and author-corrected version of record of the manuscript is available on our site with no access controls, subscription charges or pay-per-view fees (http://bmjopen.bmj.com).

If you have any questions on BMJ Open's open peer review process please email info.bmjopen@bmj.com

BMJ Open

Open-label placebo rationale revisited: A thematic analysis of lay perspectives

Journal:	*BMJ Open*
Manuscript ID	bmjopen-2021-053346
Article Type:	Original research
Date Submitted by the Author:	10-May-2021
Complete List of Authors:	Locher, Cosima; University Hospital Zurich, Department of Consultation-Liaison Psychiatry and Psychosomatics; University of Basel, Faculty of Psychology<br>Buergler, Sarah; University of Basel<br>Kost, Linda; University of Basel<br>Blease, Charlotte; Harvard Medical School, General Medicine and Primary Care<br>Frey Nascimento, Antje; University of Basel, Faculty of Psychology<br>Gaab, Jens; University of Basel
Keywords:	QUALITATIVE RESEARCH, Quality in health care < HEALTH SERVICES ADMINISTRATION & MANAGEMENT, MENTAL HEALTH, PAIN MANAGEMENT, PRIMARY CARE

SCHOLARONE™
Manuscripts

I, the Submitting Author has the right to grant and does grant on behalf of all authors of the Work (as defined in the below author licence), an exclusive licence and/or a non-exclusive licence for contributions from authors who are: i) UK Crown employees; ii) where BMJ has agreed a CC-BY licence shall apply, and/or iii) in accordance with the terms applicable for US Federal Government officers or employees acting as part of their official duties; on a worldwide, perpetual, irrevocable, royalty-free basis to BMJ Publishing Group Ltd ("BMJ") its licensees and where the relevant Journal is co-owned by BMJ to the co-owners of the Journal, to publish the Work in this journal and any other BMJ products and to exploit all rights, as set out in our licence.

The Submitting Author accepts and understands that any supply made under these terms is made by BMJ to the Submitting Author unless you are acting as an employee on behalf of your employer or a postgraduate student of an affiliated institution which is paying any applicable article publishing charge ("APC") for Open Access articles. Where the Submitting Author wishes to make the Work available on an Open Access basis (and intends to pay the relevant APC), the terms of reuse of such Open Access shall be governed by a Creative Commons licence – details of these licences and which Creative Commons licence will apply to this Work are set out in our licence referred to above.

Other than as permitted in any relevant BMJ Author's Self Archiving Policies, I confirm this Work has not been accepted for publication elsewhere, is not being considered for publication elsewhere and does not duplicate material already published. I confirm all authors consent to publication of this Work and authorise the granting of this licence.

For peer review only

Open-label placebo rationale revisited: A thematic analysis of lay perspectives

Cosima Locher[1,2,3], Sarah Buergler[3], Antje Frey Nascimento[3], Linda Kost[3], Charlotte Blease[4], & Jens Gaab[3]

[1] Department of Consultation-Liaison Psychiatry and Psychosomatic Medicine, University Hospital Zurich, Switzerland

[2] Faculty of Health, University of Plymouth, Plymouth, UK

[3] Division of Clinical Psychology and Psychotherapy, Faculty of Psychology, University of Basel, Switzerland

[4] General Medicine and Primary Care, Beth Israel Deaconess Medical Center, Harvard Medical School, Boston, USA

Address for correspondence: Cosima Locher, PhD, Department of Consultation-Liaison Psychiatry and Psychosomatic Medicine, University Hospital Zurich, Switzerland. Email: cosimaantoinette.locher@uzh.ch Phone: 0041 79 461 32 54

Keywords: open-label placebo, rationale, mechanisms, qualitative, thematic analysis

Short title: Open-label placebo rationale revisited

Word count: 5995

For peer review only

Abstract

Objectives: To analyze participants' concepts about the open-label placebo (OLP) effect; to explore participants' views about the discussion points that are applied in conventional OLP trials; and to examine participants' experiences of taking part in an OLP trial.

Design: A qualitative study using thematic analysis of semi-structured interviews.

Participants: Thirty participants that took part in a randomized controlled trial that investigated experimental OLP analgesia.

Results: Participants mostly conceptualized placebo as something that is inert and requires deception in order to be effective. Interviewees used a broad definition of placebos, going beyond a conventional notion of sugar pills. In contrast to the conventional OLP rationale, participants seldom emphasized classical conditioning as a mechanism of placebo effects, stressing a variety of other well-established components through which placebos might be therapeutic. Whereas the conventional OLP disclosures state that "a positive attitude helps but is not necessary", participants in our study applied other attitudes, such as "it's worth a try". When asked about their experiences during the trial, the majority emphasized that the concept of OLP was completely novel to them. Participants were rather skeptical about the efficacy of the intervention.

Conclusion: Integrating lay perspectives into the scientific rationale of OLP treatments might enhance the plausibility and credibility of the rationale in ethical treatments.

For peer review only

Strenghts and limitations of this study

- Despite the increasing number of OLP trials, this is, to the best of our knowledge, the first qualitative study that examined participants' attitudes about OLP
- The semi-structured interviews generated rich, contextual data
- The results contribute to the discussion regarding the use and framing of the conventional OLP treatment rationale
- The sample consisted of young university students which may limit generalizability

Additional statements

Twitter: @the_pain_net

Acknowledgments: The authors would like to thank Xenia Knüsel and Sebastian Hasler for their assistance with the data transcription. They would also like to thank Berfin Bakis for her help with the translation of the quotations. We also thank Mareike Rytz for her help with the references.

Contributors: CL, CB, and JG: study concept and design; CL: conducted all interviews and drafted the manuscript; SH and XK: transcribed all interviews; CL, SB, AF, and LK: coded all interviews; CB, JG, SB, AF, and LK: contributed to critical advice and revisions of the manuscript. All authors read and approved the final manuscript.

Funding statement: CL received funding from the Swiss National Science Foundation (SNSF): P4P4PS_194536.

Competing interests statement: The authors have no conflicts of interest to disclose.

Data availability statement: Anonymized transcripts of the qualitative interviews are stored at the *Harvard Dataverse* data repository: https://doi.org/10.7910/DVN/ET5PVQ

ORCID ID: Cosima Locher: https://orcid.org/0000-0002-9660-0590

Patient and public involvement: Patients and the public were not involved in the design of the study; however, the study specifically focuses on participant perceptions.

For peer review only

Introduction

There is evidence that placebos exhibit substantial effects with promising clinical potential in various mental and physical complaints [1]. Nevertheless, the potential of placebo effects in clinical practice is impeded as its administration requires deception. Some health researchers have argued that deceptive placebos may sometimes be ethical in the interests of therapeutic gain – for example, where no other treatment options are available, or on the grounds that the deception is trivial and does not involve a major threat to patient autonomy [2,3]. However, most ethicists argue that deception in clinical context is unjustified and violates duties to be honest with patients [4,5]. In addition, deception may risk derailing trust in clinicians, leading to possible harms including disengagement with healthcare [6,7].

Here, openly prescribed placebos offer the possibility to harness placebo effects in a transparent and thus ethical way [8]. The OLP approach has been examined in various physical and mental disorders, e.g., chronic low back pain [9,10], irritable bowel syndrome [11], allergic rhinitis [12–14], ADHD [15,16], and fatigue among cancer survivors [17,18]. So far, two meta-analysis examining OLPs revealed that patients in an OLP condition exhibit significantly greater improvement than those in a control group [19,20]. There is evidence that OLPs may have significant effects on sub-clinical conditions, e.g., menopausal hot flushes [21], and test anxiety [22,23], as well as on well-being, e.g., sleep quality [24], and wound healing [25]. OLP has also been investigated in experimental settings such as experimentally induced pain, and allergic reactions [26–29].

All of these studies clearly inform participants that the intervention consists of “honestly described placebo pills” [30], applying a plausible and positive rationale. A standard approach has emerged with these studies, whereby disclosure about the rationale conveys four discussion

For peer review only

points, which are based on both, study findings and clinical considerations [11,30]: (1) *Placebos are powerful*. The major aim of this statement is to remove the stigma of placebo effects and to openly inform participants about the wide array of study findings that report powerful placebo effects in double-blind randomized controlled trials (RCTs) [30]. (2) *The body can automatically respond to taking placebo pills like Pavlov's dogs who salivated when they heard a bell*. The second discussion point emphasizes classical conditioning as a major mechanism of how placebos might elicit therapeutic effects [31,32]. (3) *A positive attitude helps but is not necessary*. The aim of the third key point may be designed to take participants' skepticism seriously and to emphasize that they do not have to believe in the treatment for it to work [30]. (4) *Taking the pills faithfully is critical*. The final discussion point is related to study findings from RCTs that reveal that adherence is crucial for a (medical) treatment to work [33,34]. In this paper, we describe OLP studies that focus on these four discussion points as "conventional OLP" disclosures.

From an ethical point of view, transparency in the OLP rationale is crucial in order to respect participants' autonomy [6,35]. So far, two qualitative investigations have examined what participants experience when they are informed that they were assigned to the placebo arm in a placebo-controlled clinical trial [36,37]. These studies reveal that participants hesitate and doubt whether the placebo treatment has a potential to heal, yet many patients also spontaneously express hope [36]. Although informative, these findings are not transferable to the field of OLP, where participants know that they will receive a placebo. There is only one pilot study that examined participants' perspectives on the OLP treatment [38]. In that study, 10 participants were asked about their definition of placebo, whether the OLP rationale was clear to them, and

For peer review only

whether anything about the study was misleading. None of the questions, however, solicited feedback on attitudes about OLP.

Therefore, this qualitative study set out to investigate participants' attitudes about (open-label) placebo. The study was nested within a RCT that investigated OLP analgesia in healthy participants. The aims of the study were threefold: First, to analyze participants' concepts about the (open-label) placebo effect. Second, to compare participants' views on OLP with the four discussion points that are applied in conventional OLP trials. Third, to summarize participants' experiences of taking part in an OLP trial. For all our aims, we strove to compare participants' statements of the three intervention groups of the quantitative study, i.e., open-label without rationale (OPR-), open-label placebo with rationale (OPR+), and deceptive placebo (DP) groups.

Methods

Study design

This is a mixed methods qualitative study embedded within a RCT investigating the OLP treatment in healthy participants [28]. The study applied an experimental heat pain paradigm, comparing four groups, i.e., no treatment (NT), OPR-, OPR+, and DP. Semi-structured interviews were scheduled at the end of each participant's treatment. The study was approved by the Local Ethics Committee of the Canton Basel, Switzerland (EKNZ 2015-246).

Study participants

Thirty participants from the intervention groups (i.e., OPR-, OPR+, and DP) were randomly selected to take part in the nested qualitative study. Interviewing participants from the different intervention groups allowed us to examine attitudes towards the OLP approach in dependence on previous experimental experiences. Table 1 shows participants' basic demographic information.

For peer review only

141 Recruitment started in January 2016 and lasted until July 2016. Written informed consent was
142 obtained from each participant.

143 **Interview procedure**

144 The interviews were conducted by a trained interviewer (CL) applying a semi-standardized
145 interview guide. All participants have been interviewed about their attitudes towards the (open-
146 label) placebo effect. In order to ensure that participants who did not receive the OLP treatment
147 (i.e., OPR- and DP groups) are not primed by the existing open-label placebo rationale, we
148 aimed to explore their attitudes towards the placebo effect with a bottom-up approach. The
149 interview covered the following components: (a) identity of the placebo effect, (b) possible
150 causes of the placebo effect, (c) opinions about short- and long-term consequences of the placebo
151 effect, (d) the perceived control over the placebo effect and (e) the timeline of the placebo effect.
152 A broad interview guide was prepared comprising 21 question as a tool to obtain information
153 (see eAppendix 1). Interviews lasted between 21 and 66 minutes. The interviews were audio-
154 taped and verbatim transcripts were written. The listed quotations have been translated from
155 German to English.

156 The first author who conducted the interviews (CL) completed her PhD in 2017. In her research,
157 she focuses on the effects, mechanisms, and routes of administration of (open-label) placebo. For
158 that, she applies different methodological approaches, encompassing qualitative studies,
159 experimental trials, clinical investigations, and (network) meta-analyses.

160 **Qualitative Analysis**

161 For data analysis, MAXQDA 2020 (VERBI Software, 2019) was used [39]. We followed an
162 inductive-deductive hybrid approach [40] and incorporated primarily an inductive data-driven
163 approach to allow the examination of core themes for a phenomenon with limited existing theory

For peer review only - http://bmjopen.bmj.com/site/about/guidelines.xhtml

or research literature [41–43], complemented by a deductive ideation of the theoretical points of the OLP treatment rationale [44]. With the aim to describe common patterns across the interviews, the following six steps were performed [45]: First, the comment transcripts were read 3-4 times by four coders (SB, LK, AF, and CL) in order to achieve familiarization with the data. Second, the two main coders (SB and LK) identified brief descriptive labels ('codes') that represented different aspects of interviewees' descriptions. More than a single code was applied if quotations had multiple meanings. Third, the four coders (SB, LK, AF, and CL) examined the codes and sorted them into different themes with shared meaning units. Fourth, the themes were reviewed, i.e., themes that were not supported by the data were modified by the coders. Fifth, the coders developed a detailed analysis of each theme by defining final names. Finally, all authors took part in authoring the manuscript. We sought analytical rigor by involving multiple coders.

Results

Participants

Participants had a mean age of 27.2 (SD 9.73) years, and 63% of the participants were female (see Table 1). The three groups were comparable regarding age ($F(2,27) = 1.09$, $P = 0.35$), sex ($X^2(2) = 0.29$, $P = 0.866$), family status ($X^2(4) = 3.61$, $P = 0.462$), educational level ($X^2(6) = 3.00$, $P = 0.809$), and employment level ($X^2(4) = 3.49$, $P = 0.479$). Quantitative results were reported elsewhere [28,46]. The majority of participants assigned to the OPR- and OPR+ groups believed they were receiving a placebo cream (17/20). Most participants in the deceptive placebo group also believed they were receiving the analgesic cream (6/10).

Overview

In the 30 interviews, 667 text passages of 30'379 words were identified and summarized into 65 categories. These categories were subsumed in 8 main categories, which were classified in three

For peer review only

superordinate categories: (1) the placebo concept; (2) open-label placebo rationale; and (3) experiences of taking part in an open-label placebo trial (see Table 2). An overview of all superordinate categories, main categories and categories is provided in the supplement, also revealing the number of quotations for each category per group (eTable 1). In the following, we present the four superordinate categories and main categories. For the complete list of categories please see Table 2.

1. Placebo concept

1.1 Descriptive

The vast majority of participants agreed on the definition of placebo as something that is characterized by its inertness and its deceptive administration. This assumption was independent of group allocation; for example:

- *"To me, a placebo is a drug without actual ingredients"* (DP; participant 42)

Likewise, many participants emphasized that deception is a prerequisite for placebos to work. Notably, examples not only included the classical placebo pill, yet also deceptive aspects in politics and advertisements that lead to a change of belief or behavior; for example:

- *"Well, in term of deception - so when you use the term deception, I don't know, in politics that happens; I often feel like you can achieve something with deceptive means"* (OPR-; participant 89)

A few comments linked the placebo concept to the design and conductance of clinical studies, such as randomized controlled trials; for example:

- *"Well, I've heard that quite often now, that, I don't know, experiments or other studies were conducted where people were given placebos"* (OPR-; participant 56)

For peer review only

Notably, two participants from the OPR- group expressed the view that placebos are like a copy or a substitute of a verum intervention, which is, however, less powerful:

- *"Yes, some kind of substitute, like - just like this clone phenomenon, that is kind of like a copy but not quite the same, and also not as good <<laughs>>"* (OPR-; participant 135)

1.2. Effectiveness

Interviewees expressed a variety of perspectives about the effectiveness of placebos. Especially participants who were allocated to the OPR+ and OPR- groups emphasized the paradox that placebos are inert, yet reveal positive effects; for example:

- *"When I think of a 'drug' it's a drug that doesn't have any medical ingredients; it's like neutral actually yet triggers an effect afterwards"* (OPR+; participant 141)

Similarly, many participants indicated general agreement that the intake of placebos has an effect on the body. This was independent of group allocation; for example:

- *"[Mhm] (.) more than an imagination actually, with actual physical effects"* (OPR+; participant 109)

In opposition to this view, other interviewees neglected that placebos can have a physical effect. Some interviewees stressed that placebos are genuinely free of effect, also using terms like "sham" and "fake". Notably, except one participant, all statements stemmed from participants assigned to the OPR- or DP groups; for example:

- *"No no no, it's really only controlled by the mind in our head, so one should not be able to measure anything purely physically"*(OPR-; participant 89)

1.3. Specific examples

Building on this theme, the vast majority of interviewees offered a variety of examples of, what they considered to be, placebos. Examples went beyond the classical pill encompassing

For peer review only

autosuggestion, esotericism, complementary medicine, food supplements, advertisement, mindfulness, and food. Notably, patients allocated to the OPR+ group gave more context to these statements, explaining why they think that these kinds of placebos might work:

- *"Yes, it probably is the case that sometimes, when I notice that I'm getting a cold or have a cold, I just take and eat ginger, and I'm convinced that it works every single time. In this case I also feel like it works <<laughs>>; and I can certainly imagine that it just…that these rituals also have an effect, yes"* (OPR+; participant 157)

More broadly, and often related to daily life experiences, interviewees also mentioned that a placebo can be a general act, an activity, or something that happens in the interpersonal contact; for example:

- *"The sauna, for example, I do it regularly as soon as winter starts. And was never sick ever since"* (OPR-; participant 74)
- *"I always watch TV in order to fall asleep. I fall asleep faster when I do that than when I don't. A placebo is also like a ritual, yes"* (OPR+; participant 146)

2. Open-label placebo rationale

All interviewees stressed components that can be linked to the established OLP rationale. Whereas some aspects were in line with the discussion points of the rationale, others were complementary or even contradictory:

2.1 First discussion point

The majority of interviewees concretized the anticipated effect of an OLP treatment, which is related to the first discussion point of the OLP rationale (i.e., *placebos are powerful*).

For peer review only

Participants agreed that an OLP treatment is less effective and/or has shorter lived effects than the deceptive placebo treatment; for example:

- *"But I feel like if you know that it's a placebo, it may not be as long-lasting than if you don't"* (OPR+; participant 146)

More precisely, interviewees stated that key mechanisms such as positive expectations and belief systems are reduced in the OLP treatment. It was also argued that the lack of deception is associated with a significant reduction of placebo efficacy. Most concerns were expressed from participants assigned to the OPR- and DP group with the exception of two people from the OPR+ group; for example:

- *"[Hmm] well, I believe the whole belief system collapses a little bit; I mean when you have a very medical name and an appealing packaging, it will work even if there's a placebo pill inside"* (OPR-; participant 74)
- *"For me a placebo is something that is really connected to a strong belief, so I have a feeling that it only works, or especially works, when for example one doesn't know that it is a placebo"* (OPR+; participant 141)

Some interviewees from the OPR+ group, however, believed that an OLP treatment can be powerful; for example:

- *"Yes, even if it's just a placebo, uhm, simply from the studies that have been conducted so far, one knows that it can work; and already that, that statement has had an effect on me and I thought 'well okay so even if it's open it still works a little bit' so"* (OPR+; participant 35)

2.2 Second discussion point

For peer review only

Interviewees listed a big variety of mechanisms that make the OLP treatment work. In line with the second discussion point of the established OLP rationale (i.e., *the body can automatically respond to taking placebo pills like Pavlov's dogs who salivated when they heard a bell)*, some interviewees emphasized classical conditioning as a key mechanism for placebos to be effective. Notably, only participants from the OPR+ group explicitly referred to the term 'conditioning' whereas others talked about the impact of experiences and unconscious processes; for example:

- *"Yes, because we're used to it, just like for example when I saw the drug and had the package in my hand, yes for me it's association so it's a drug, only drugs look like that"* (OPR+; participant 35)

Notably, however, comments that could be linked to 'conditioning' represented only a minority of interviewees' statements. The vast majority highlighted the impact of personality traits such as openness and a positive attitude. This was independent of group allocation; for example:

- *"I think I'm pretty open-minded regarding new things, maybe that has something to do with it, too"* (OPR+; participant 141)

Besides people's personality components and general attitudes, interviewees also stressed personal mindsets that are directed towards the future, encompassing positive expectancies, beliefs and hope; for example:

- *"[Hmm] or from the expectations one might have; I can imagine that simply through the doctor administering something, like a preparation, people will generate certain expectations"* (OPR+; participant 109)

More broadly, participants acknowledged the mind-body connection and psychological components in general; for example:

For peer review only

- *"Well, it's not that something just happens by mind power, but something in my body changes through mind power. So, there's definitely something happening on a molecular basis, that's clear" (*DP; participant 154)

Besides components that are linked to the individual patient, other comments underlined the impact of a trust-based patient-provider relationship. Again, this was independent of group allocation; for example:

- *"So, I took it when I was a kid because my doctor recommended it to me, and I mean as a kid you trust doctors 100% anyway, right? <<laughs>>and I've always felt very comfortable in that practice, that's also very important. And the investigator [of the study] was very nice and always explained exactly what she was doing, I think that there's a big connection" (*OPR-; participant 99)
- *"Absolutely, so I had one [an investigator] who had long conversations with me, and consequently one is- you're more easily inclined to engage in something when you feel like you're in good hands and he really took his time too so, yeah" (*DP; participant 48)

Some interviewees highlighted other placebo mechanisms such as the power of imagination and the ritual. Notably, one participant also emphasized that a response to receiving a placebo might also encompass the natural course of the disease:

- *"So, in a first step someone takes a placebo, and by pure chance their headache fades away. Well not in that exact moment, but after a couple of minutes, on its own, right? [interviewer: like a natural course?] Exactly. So, the pain comes and goes without taking any painkillers"* (OPR-; participant 39)

2.3 Third discussion point

For peer review only

The third discussion point of the original OLP rationale (i.e., *a positive attitude helps but is not necessary*) has the general aim to take participants' skepticism seriously and to operate as a kind of relativization. Crucially, none of the participants spontaneously expressed such a thought. However, they referred to other aspects of relativization that would encourage them to take an OLP, such as the idea of "nothing to lose" and "it's worth to give it a try"; for example:

- *"I'd probably do it in a way where I would give the cream a chance and say, 'it doesn't do me any harm, just give it a try.'"* (OPR-; participant 89)
- *"You actually can't lose anything at all, that's another aspect of it, I think. It's definitely worth a try"* (OPR+; participant 109)
- *"Well, if it helps and doesn't harm my body in any way, then that's great"* (DP; participant 138)

Others agreed that the OLP treatment would be an option for them *if* there would be no alternative; for example:

- *"Good question, I think it depends on the situation, like how alarming it is. If I wouldn't have anything but the placebo cream, I'd probably use it <<laughs>>"* (OPR-; participant 135)

On a broader scale, interviewees speculated that the readiness to take an OLP treatment also depends on the context and the respective disorders; for example:

- *"As I said, you need to differentiate of course, as there are different forms of diseases; when I talk about the common cold or cancer therapy, those are clearly two different things, and of course you have to differentiate"* (DP; participant 138)

3. Experiences of taking part in an open-label placebo trial

For peer review only

3.1. Reaction to (open-label) placebo

The vast majority of participants assigned to the OPR+ group emphasized that the OLP approach was completely novel to them; for example:

- *"Um so I didn't know that there's research - well that there's research showing that there are effects when you know that it's a placebo and it still has an effect"* (OPR+; participant 146)

Furthermore, patients that were allocated to the OPR+ group agreed that the OLP treatment was only plausible because of the rationale that was provided during the experiment; for example:

- *"How should I say, that there's still the release of some substances; now I'm not very familiar with this <<laughs>>. But uhm I think when she [the investigator] told me about it, that it's like conditioned, that seemed plausible to me"* (OPR+; participant 141)

Opposing to this, a dominant theme in the OPR- group was the conviction that the OLP treatment is a little strange, crazy and incomprehensible; for example:

- *"But that's not negative at all, if anything I think it's a funny farce << laughs >>"* (OPR-; participant 135)
- *"It is very peculiar"* (OPR-; participant 74)

Interestingly, three participants (i.e., two from the OPR- group and one from the OPR+ group) stated that they were speculating during the experiment whether they really received a placebo; for example:

- *"You might also think 'ah maybe it's not a placebo, maybe it's real cream and they won't tell me just to see- maybe to see how I react when I don't know that it contains active ingredients, and then has an effect anyway'"* (OPR-; participant 57)

3.2. Efficacy and effectiveness during the experiment

For peer review only

Finally, interviewees also made estimates about the efficacy of placebos in the experiment. The majority of participants of the OPR+ and OPR- groups suggested that the intervention was probably not effective. Some participants from the DP group shared this impression; for example:

- *"No, I don't think so << both laugh >>, I don't think it worked, I even checked: it [the skin] was red right away, so yeah, I don't think it really worked"* (OPR-; participant 57)
- *"To be honest, I don't think it made a difference"* (OPR+; participant 34)
- *"I did not notice a difference"* (DP; participant 75)

On the contrary, some participants of the DP group reported that the intervention had an effect on their pain levels; likewise, some interviewees from the OPR+ and OPR- groups suggested that the intervention had an effect on their mind; for example:

- *"I really think, psychologically, because I um was told that with open placebos, well, the trials where people knew it's a placebo, that it worked there. I think it was through that statement actually, yes"* (OPR+; participant 35)

Some participants were also unsure about whether the intervention had an effect or not; for example:

- *"Well, I was wondering if it had an effect on my pain threshold, I don't know the results. And um, I tried to convince myself that it [the placebo] doesn't have an effect, I should be on the same threshold level [in the second round], yet there was this little thought 'it could still be effective'"* (OPR-; participant 89)

Notably, some interviewees reported that experimental components such as the cooling of the cream or the patch position had an influence on their pain experience; for example:

For peer review only

- *"But maybe I just thought um, just the cream, not the active ingredient is having an effect, just the layer << laughs >> between the plate and the skin"* (OPR-; participant 89)

Discussion

This qualitative study set out to evaluate participants' concepts about the (open-label) placebo effect, to compare participants' attitudes towards (open-label) placebos with the four discussion points of the conventional OLP disclosures, and to summarize participants' experiences during the experimental OLP trial. In the quantitative part of the study that has been published elsewhere [28], we found that the OPR+ group exhibited a significant reduction of subjective heat pain ratings that did not differ from the DP group. The present study is one of the first qualitative studies that is nested within an OLP trial.

We found that the vast majority of participants describe placebos as an intervention that is medically inert with respect to its ingredients for treating symptoms but efficacious and powerful. This is also in line with a pilot study that examined ten patients who received OLPs as an adjunctive treatment to opioids for acute pain. There, the majority of participants described placebos as something that is inactive [38]. To define a placebo as an inert substance that has no effect on human physiology is common, yet questionable. Several researchers argued that no substance is inert if "inert" is defined as something that has no physiological effect [47–49]. Inertness may also be associated with something that is neutral or unspecific. Placebos, however, reveal specific effects and mechanisms and can thus be conceptualized as a specific agent [50]. These contrasting views raise the question of whether it would be helpful to inform participants in OLP trials that placebos are specific by their nature. Another component that has been traditionally linked with the placebo term and that has been raised by the participants of our

For peer review only

For peer review only - http://bmjopen.bmj.com/site/about/guidelines.xhtml

study is the assumption that placebos need deception in order to work. Particularly in clinical practice, however, it is clearly unethical to deceive patients by prescribing placebos as deception violates the principle of respect for patient autonomy [51]. In contrast, our results reveal that the long-time association of placebo and deception may be common in the general population. This implies that the information given in OLP trials, i.e., that placebos can also work when delivered openly, is a crucial component of the OLP treatment. Interviewees described several kinds of placebos, going beyond the classical sugar pill. Notably, the spectrum of examples encompassed several aspects that have been studied in placebo research. The power of autosuggestion, for example, has been explained by a change of mind-set and a reappraisal of a situation [52]. Likewise, the practice of mindfulness has been supposed to modify mind-sets and has shown to be related to positive health outcomes [53]. Interviewees also related supportive interpersonal aspects to placebo effects, which is in line with research indicating that the alliance between the treatment provider and treatment recipient is related to the effects of the respective treatment, even if this treatment is placebo [54–57]. Finally, as outlined by some participants, religiosity and spirituality are related to hope, positive thinking, and meaning-making, all mechanisms that have been suggested to operate in placebos [e.g., 58]. Taken together, interviewees spontaneously outlined several kinds of placebos that have been the subject of multiple research approaches; this is in line with recent attempts to generalize placebos and to study them outside the medical context [e.g., 6].

The statements given by the interviewees were further compared with the conventional OLP rationale. Interviewees did not explicitly engage with the first discussion point (i.e., *placebos are powerful*) despite interviewer attempts in the OPR+ group, i.e., most made no statements in relation to the effectiveness of placebos, yet agreed that *openly* prescribed placebos

For peer review only

might be less effective than deceptive placebos. This is in contrast to some first indications showing that openly described placebos work as well as deceptive placebos [28,59]. In order to inform patients that honestly prescribed placebos are also powerful, newer OLP trials started to outline the findings from the first controlled OLP trial [11], usually showing a video sequence of an American NBC news report of the respective study [e.g., 9]. Our findings indicate that it is of utmost importance to inform participants about the promising evidence of OLP trials.

In relation to the second discussion point (i.e., *the body can automatically respond to taking placebo pills like Pavlov's dogs*), which clearly refers to one of the most important mechanisms of placebo effects (i.e., classical conditioning), quotations from our participants were surprising. We found that only one participant from the OPR+ group mentioned conditioning. Evidence in the field of conditioning also suggests that behavior cannot only be triggered by verbal information, yet also by nonconscious stimuli, a differentiation that was not mentioned by our participants. Besides classical conditioning, the second most dominant conceptual model to explain placebo effects is expectancy [60,61], a mechanism that was frequently stressed by our interviewees, also in relation to general beliefs and habits. A third mechanism that has received considerable attention and has shown to induce placebo effects is the patient-provider relationship [62], a component that was also emphasized by many of our interviewees, especially in relation to trust. Notably, participants of our study stressed a wide array of other mechanisms through which placebos work, including general attitudes, beliefs, hope, the ritual, and the power of imagination. All these factors are established mechanisms in placebo research [for an overview, see e.g., 63], usually subsumed under external and internal elements of the treatment context [64]. Another psychological component that received less attention from placebo researchers and that was frequently addressed by our participants is hope.

For peer review only

This is in line with other qualitative placebo (yet not openly prescribed placebo) studies reporting that patients spontaneously expressed hope, which they usually explicitly distinguish from expectation [36,65,66]. Whereas expectancy is linked to cognitive states and a prediction of the future, hope is not so much a prediction but rather more an existential stance [36]. Taken together, our findings reveal that participants of an OLP trial intuitively list a wide array of established placebo mechanisms. This raises the question why classical conditioning is *exclusively* stressed by the conventional OLP disclosures. A recent survey study that examined participants' preferences of placebo explanations found that other mechanisms than classical conditioning (i.e., positive expectations and brain mechanisms) were rated most preferable [67]. Mechanisms that have been shown to be relevant for the efficacy of placebos cannot be transferred to the field of openly prescribed placebos. It has been argued that patient expectations about the efficacy of a treatment substantially differ between conventional and open-label placebo approaches [27]. Thus, it might be worth considering informing participants in OLP trials about the variety of mechanisms that operate in *openly* prescribed placebos. This will ensure that the provided information is based on newest evidence.

Finally, the third discussion point of the conventional OLP rationale (i.e., *a positive attitude helps but is not necessary*) aims to reduce skepticism and works as a kind of relativization. Although interviewees of our study expressed different strategies to reinforce realistic (and not too high) expectations, none of them used the reasoning that *a positive attitude helps but is not necessary* or comparable relativization strategies. Interviewees argued that "it's worth a try"; "if it helps and does no harm", or "worth to see what happens". These viewpoints are not new: patients in randomized, sham-controlled trials who do not know whether they are assigned to the verum or the sham condition often argue that it is worth giving it a try [36,37,68].

For peer review only

484 The underlying heuristic appears to be: "I lose little if the placebo does not work, but gain a lot if
485 it does work" [69]. Notably, some participants also outlined that they would take a placebo "if
486 it's the only option". This is related to the notion that their openness would "depend on the
487 symptoms or disorder", a finding that is in line with a previous qualitative study, applying a
488 scenario to explain OLP to participants [70]. Patients with chronic primary pain, for example,
489 often describe that different treatment approaches have turned out to be unsatisfactory, leading to
490 a perceived lack of alternatives and a state of demoralization [71], which might lead to an
491 openness towards OLPs. Therefore, the idea of informing participants that *a positive attitude*
492 *helps but is not necessary* might be problematic: not only do participants use other strategies to
493 reduce skepticism, they are also convinced that positive attitudes are an important mechanism for
494 placebos to work – a sentiment which is supported by current scientific evidence [72].

495 Finally, we were interested in participants' experiences in an experimental OLP trial. We
496 found that for the vast majority of interviewees, the OLP approach was completely novel. Most
497 interviewees had the impression that the treatment was non-effective. Slightly more participants
498 from the DP group stated that the efficacy of the intervention was possible or even likely.
499 Especially participants assigned to the OPR+ group mentioned that the efficacy of the
500 intervention was "unclear". Interestingly, slightly more participants from the OPR- group stated
501 that the efficacy of the intervention is "possible" when compared to the OPR+ group. These
502 rather more skeptical and reserved expressions related to the efficacy of the intervention, that we
503 found independent of group assignment, are in contrast to results of the original RCT. There,
504 groups with a rationale (OPR+ and DP) reported significantly lower heat pain intensity and
505 unpleasantness ratings than the OPR- group [28]. Beyond the presumed efficacy of the
506 intervention, there were some group-dependent reactions to the concept of OLP. Whereas

For peer review only - http://bmjopen.bmj.com/site/about/guidelines.xhtml

participants from the OPR+ group stressed that the plausible rationale was probably the most important component of the treatment, participants from the OPR- group said that the intervention was "strange, crazy, and incomprehensible". The differing views of these two groups underscore the importance of a plausible explanation of the concept of OLPs to counter possible skepticism.

Strengths and limitations

The present qualitative study has a number of limitations. The OLP trial recruited young university students, and this may limit generalizability to other demographic populations. The participants were well educated and informed about placebos, which was reflected in the sophisticated answers of the participants (e.g., only one participant was not aware of the term "placebo"). A recent survey study also found that a higher education is related to more placebo knowledge [67]. Second, the present qualitative study examined healthy participants with presumably lower motivation for pain relief than patients. Likewise, the study did not investigate attitudes of participants toward practitioners who offer OLPs, or about possible negative effects, such as feelings of stigmatization. Some patient populations may be highly resistant to being offered OLPs or offended by the idea. Third, although we did set out to compare participants' views of OLPs with the conventional OLP disclosures, we were not able to make conclusions about the fourth discussion point (i.e., *taking the pills faithfully is critical*) since the topic of adherence was not applicable in our experimental RCT. Fourth, the researcher conducting all interviews was not blind to the group allocation, probably leading to a bias in the framing of the questions. Therefore, we decided that the two main coders were neither researchers who delivered the treatment nor those who conducted the interviews. Despite these limitations, this qualitative study offers a novel perspective on participants' views about openly prescribed

For peer review only

placebos and contributes to the discussion regarding the use and framing of the OLP treatment rationale. The qualitative approach that we chose to reflect on the conventional OLP disclosures is one possibility. Other ways to further make suggestions for the adaption of the rationale might be to integrate newest evidence-based findings of OLP trials or to focus on ethical considerations.

Conclusion

Participants of our experimental OLP trial spontaneously expressed a wide array of placebo definitions, examples, and underlying mechanisms. In their descriptions, they went beyond theoretical speculations and provided a variety of personal experiences. Given the big variety of conceptualizations, but upholding standards of evidence, clinical practice might profit from tailoring the provided information to the specific needs of individual patients, which is in line with the recommendations formulated by a placebo expert group [73]. We were able to link participants' quotations to the conventional OLP rationale. Overall, we propose that the OLP rationale would gain from further adaptions and should be revisited. We suggest that participants of OLP trials should be informed of *multiple* placebo mechanisms that exist, including but not limited to classical conditioning. Conceivably, this might further increase the plausibility of the rationale and will also be in line with newest evidence. Furthermore, and in order to set realistic expectations, we propose that participants should be motivated to "just give it a try". This is in contrast to the current strategy of the rationale to decrease possible skepticism. Further research should move beyond conceptualizations of placebos, and opinions about their effectiveness, to attitudes about the acceptability of being offered OLPs, especially for patients living with a variety of placebo-responsive symptoms. Such studies should explore

For peer review only

perceptions about the competence of clinicians who offer OLPs. We also recommend that future research solicits the views of patients drawn from different demographic populations.

For peer review only

For peer review only - http://bmjopen.bmj.com/site/about/guidelines.xhtml

References

1 Enck P, Bingel U, Schedlowski M, *et al.* The placebo response in medicine: minimize, maximize or personalize? *Nat Rev Drug Discov* 2013;**12**:191–204. doi:10.1038/nrd3923

2 Foddy B. A Duty to Deceive: Placebos in Clinical Practice. *Am J Bioeth* 2009;**9**:4–12. doi:10.1080/15265160903318350

3 O'Neill O. Paternalism and partial autonomy. *J Med Ethics* 1984;**10**:173–8. doi:10.1136/jme.10.4.173

4 Blease CR. The principle of parity: the 'placebo effect' and physician communication. *J Med Ethics* 2012;**38**:199–203. doi:10.1136/medethics-2011-100177

5 Bok S. The Ethics of Giving Placebos. *Sci Am* 1974;**231**:17–23. doi:10.1038/scientificamerican1174-17

6 Gaab J, Blease C, Locher C, *et al.* Go open: A plea for transparency in psychotherapy. *Psychol Conscious (Wash D C)* 2016;**3**:175–98. doi:doi.org/10.1037/cns0000063

7 Schöne-Seifert B. Placebos and placeboids in clinical practice: conceptual and ethical considerations. *Nervenarzt* 2020;**91**:684–90. doi:10.1007/s00115-020-00943-8

8 Blease CR, Bernstein MH, Locher C. Open-label placebo clinical trials: is it the rationale, the interaction or the pill? *BMJ Evid Based Med* 2019;**25**:159–65. doi:10.1136/bmjebm-2019-111209

9 Carvalho C, Caetano JM, Cunha L, *et al.* Open-label placebo treatment in chronic low back pain: A randomized controlled trial. *Pain* 2016;**157**:2766–72. doi:10.1097/j.pain.0000000000000700

10 Kleine-Borgmann J, Schmidt K, Hellmann A, *et al.* Effects of open-label placebo on pain, functional disability, and spine mobility in patients with chronic back pain: a randomized controlled trial. *Pain* 2019;**160**:2891–7. doi:10.1097/j.pain.0000000000001683

11 Kaptchuk TJ, Friedlander E, Kelley JM, *et al.* Placebos without deception: a randomized controlled trial in irritable bowel syndrome. *PLoS One* 2010;**5**:e15591. doi:https://doi.org/10.1371/journal.pone.0015591

12 Kube T, Hofmann VE, Glombiewski JA, *et al.* Providing open-label placebos remotely—A randomized controlled trial in allergic rhinitis. *PLOS ONE* 2021;**16**:e0248367. doi:10.1371/journal.pone.0248367

13 Schaefer M, Harke R, Denke C. Open-Label Placebos Improve Symptoms in Allergic Rhinitis: A Randomized Controlled Trial. *Psychother Psychosom* 2016;**85**:373–4. doi:10.1159/000447242

14 Schaefer M, Sahin T, Berstecher B. Why do open-label placebos work? A randomized controlled trial of an open-label placebo induction with and without extended information about the placebo effect in allergic rhinitis. *PLoS One* 2018;**13**:e0192758. doi:10.1371/journal.pone.0192758

15 Sandler AD, Glesne CE, Bodfish JW. Conditioned placebo dose reduction: A new treatment in attention-deficit hyperactivity disorder? *J Dev Behav Pediatr* 2010;**31**:369–75. doi:10.1097/DBP.0b013e3181e121ed

16 Sandler AD, Bodfish JW. Open-label use of placebos in the treatment of ADHD: A pilot study. *Child Care Hlth Dev* 2008;**34**:104–10. doi:10.1111/j.1365-2214.2007.00797.x

17 Hoenemeyer TW, Kaptchuk TJ, Mehta TS, *et al.* Open-Label Placebo Treatment for Cancer-Related Fatigue: A Randomized-Controlled Clinical Trial. *Sci Rep* 2018;**8**:2784. doi:10.1038/s41598-018-20993-y

For peer review only

18 Zhou ES, Hall KT, Michaud AL, *et al.* Open-label placebo reduces fatigue in cancer survivors: a randomized trial. *Support Care Cancer* 2018;**27**:2179–87. doi:10.1007/s00520-018-4477-6
19 Charlesworth JEG, Petkovic G, Kelley JM, *et al.* Effects of placebos without deception compared with no treatment: A systematic review and meta-analysis. *J Evid Based Med* 2017;**10**:97–107. doi:10.1111/jebm.12251
20 von Wernsdorff M, Loef M, Tuschen-Caffier B, *et al.* Effects of open-label placebos in clinical trials: a systematic review and meta-analysis. *Sci Rep* 2021;**11**:3855. doi:10.1038/s41598-021-83148-6
21 Pan Y, Meister R, Löwe B, *et al.* Open-label placebos for menopausal hot flushes: a randomized controlled trial. *Sci Rep* 2020;**10**:20090. doi:10.1038/s41598-020-77255-z
22 Kleine-Borgmann J, Schmidt K, Billinger M, *et al.* Effects of open-label placebos on test performance and psychological well-being in healthy medical students: a randomized controlled trial. *Sci Rep* 2021;**11**:1–11. doi:10.1038/s41598-021-81502-2
23 Schaefer M, Denke C, Harke R, *et al.* Open-label placebos reduce test anxiety and improve self-management skills: A randomized-controlled trial. *Sci Rep* 2019;**9**:13317. doi:10.1038/s41598-019-49466-6
24 El Brihi J, Horne R, Faasse K. Prescribing Placebos: An Experimental Examination of the Role of Dose, Expectancies, and Adherence in Open-Label Placebo Effects. *Ann Behav Med* 2019;**53**:16–28. doi:10.1093/abm/kay011
25 Mathur A, Jarrett P, Broadbent E, *et al.* Open-label placebos for wound healing: a randomized controlled trial. *Ann Behav Med* 2018;**52**:902–8. doi:10.1093/abm/kax057
26 Disley N, Kola-Palmer S, Retzler C. A comparison of open-label and deceptive placebo analgesia in a healthy sample. *J Psychosom Res* 2021;**140**:110298. doi:10.1016/j.jpsychores.2020.110298
27 Leibowitz KA, Hardebeck EJ, Goyer JP, *et al.* The role of patient beliefs in open-label placebo effects. *Health Psychol* 2019;**38**:613–22. doi:10.1037/hea0000751
28 Locher C, Frey Nascimento A, Kirsch I, *et al.* Is the rationale more important than deception? A randomized controlled trial of open-label placebo analgesia. *Pain* 2017;**158**:2320–8. doi:10.1097/j.pain.0000000000001012
29 Meeuwis SH, van Middendorp H, van Laarhoven AIM, *et al.* Effects of Open- and Closed-Label Nocebo and Placebo Suggestions on Itch and Itch Expectations. *Front Psychol* 2019;:436. doi:10.3389/fpsyt.2019.00436
30 Kaptchuk TJ. Open-Label Placebo: Reflections on a Research Agenda. *Perspect Biol Med* 2018;**61**:311–34. doi:10.1353/pbm.2018.0045
31 Benedetti F, Pollo A, Lopiano L, *et al.* Conscious Expectation and Unconscious Conditioning in Analgesic, Motor, and Hormonal Placebo/Nocebo Responses. *J Neurosci* 2003;**23**:4315–23. doi:10.1523/JNEUROSCI.23-10-04315.2003
32 Schedlowski M, Enck P, Rief W, *et al.* Neuro-Bio-Behavioral Mechanisms of Placebo and Nocebo Responses: Implications for Clinical Trials and Clinical Practice. *Pharmacol Rev* 2015;**67**:697–730. doi:10.1124/pr.114.009423
33 Kaptchuk TJ. Placebo studies and ritual theory: a comparative analysis of Navajo, acupuncture and biomedical healing. *Philos Trans R Soc Lond B Biol Sci* 2011;**366**:1849–58. doi:10.1098/rstb.2010.0385
34 Thompson JJ, Ritenbaugh C, Nichter M. Reconsidering the Placebo Response from a Broad Anthropological Perspective. *Cult Med Psychiatry* 2009;**33**:112–52. doi:10.1007/s11013-

For peer review only

008-9122-2

35 Blease CR, Kelley JM, Trachsel M. Informed Consent in Psychotherapy: Implications of Evidence-Based Practice. *J Contemp Psychother* 2018;**48**:69–78. doi:10.1007/s10879-017-9372-9

36 Kaptchuk TJ, Shaw J, Kerr CE, *et al.* “Maybe I Made Up the Whole Thing”: Placebos and Patients’ Experiences in a Randomized Controlled Trial. *Cult Med Psychiatry* 2009;**33**:382–411. doi:10.1007/s11013-009-9141-7

37 Paterson C, Zheng Z, Xue C, *et al.* “Playing Their Parts”: The Experiences of Participants in a Randomized Sham-Controlled Acupuncture Trial. *J Altern Complement Med* 2008;**14**:199–208. doi:10.1089/acm.2007.0682

38 Bernstein MH, Magill M, Weiss A-P, *et al.* Are conditioned open placebos feasible as an adjunctive treatment to opioids? Results from a single-group dose-extender pilot study with acute pain patients. *Psychother Psychosom* 2019;**88**:380–2. doi:10.1159/000503038

39 VERBI Software. (2019). MAXQDA 2020 [computer software]. Berlin, Germany: VERBI Software. MAXQDA. 2019.maxqda.com (accessed 19 Dec 2020).

40 Fereday J, Muir-Cochrane E. Demonstrating Rigor Using Thematic Analysis: A Hybrid Approach of Inductive and Deductive Coding and Theme Development. *Int J Qual Methods* 2006;**5**:80–92. doi:10.1177/160940690600500107

41 Hsieh H-F, Shannon SE. Three Approaches to Qualitative Content Analysis. *Qual Health Res* 2005;**15**:1277–88. doi:10.1177/1049732305276687

42 Oakley A, Strange V, Bonell C, *et al.* Process evaluation in randomised controlled trials of complex interventions. *BMJ* 2006;**332**:413–6. doi:10.1136/bmj.332.7538.413

43 O’Cathain A. *A Practical Guide to Using Qualitative Research with Randomized Controlled Trials*. 1st ed. Oxford University Press 2018. https://oxfordmedicine.com/view/10.1093/med/9780198802082.001.0001/med-9780198802082

44 Elo S, Kyngäs H. The qualitative content analysis process. *J Adv Nurs* 2008;**62**:107–15. doi:https://doi.org/10.1111/j.1365-2648.2007.04569.x

45 Braun V, Clarke V. Using thematic analysis in psychology. *Qual Res Psychol* 2006;**3**:77–101. doi:10.1191/1478088706qp063oa

46 Locher C, Frey Nascimento A, Kossowsky J, *et al.* Open-label placebo response – Does optimism matter? A secondary-analysis of a randomized controlled trial. *J Psychosom Res* 2019;**116**:25–30. doi:10.1016/j.jpsychores.2018.11.009

47 Annoni M, Blease C. A Critical (and Cautiously Optimistic) Appraisal of Moerman’s “Meaning Response.” *Perspect Biol Med* 2018;**61**:379–87. doi:10.1353/pbm.2018.0050

48 Brody H. The placebo response: Recent research and implications for family medicine. *J Fam Pract* 2000;**49**:649–54.

49 Howick J. The relativity of ‘placebos’: defending a modified version of Grünbaum’s definition. *Synthese* 2017;**194**:1363–96. doi:10.1007/s11229-015-1001-0

50 Kirsch I, Wampold B, Kelley JM. Controlling for the placebo effect in psychotherapy: Noble quest or tilting at windmills? *Psychol Conscious (Wash D C)* 2016;**3**:121–31. doi:10.1037/cns0000065

51 Miller FG, Colloca L. The Legitimacy of Placebo Treatments in Clinical Practice: Evidence and Ethics. *Am J Bioeth* 2009;**9**:39–47. doi:10.1080/15265160903316263

52 Crum AJ, Langer EJ. Mind-Set Matters: Exercise and the Placebo Effect. *Psychol Sci* 2007;**18**:165–71. doi:10.1111/j.1467-9280.2007.01867.x

53 Goldberg SB, Tucker RP, Greene PA, *et al.* Mindfulness-based interventions for

For peer review only

psychiatric disorders: A systematic review and meta-analysis. *Clin Psychol Rev* 2018;**59**:52–60. doi:10.1016/j.cpr.2017.10.011
54 Birkhäuer J, Gaab J, Kossowsky J, *et al.* Trust in the health care professional and health outcome: A meta-analysis. *PLOS ONE* 2017;**12**:e0170988. doi:10.1371/journal.pone.0170988
55 Gaab J, Kossowsky J, Ehlert U, *et al.* Effects and Components of Placebos with a Psychological Treatment Rationale–Three Randomized-Controlled Studies. *Sci Rep* 2019;**9**:1421. doi:10.1038/s41598-018-37945-1
56 Kaptchuk TJ, Kelley JM, Conboy LA, *et al.* Components of placebo effect: randomised controlled trial in patients with irritable bowel syndrome. *BMJ* 2008;**336**:999–1003. doi:10.1136/bmj.39524.439618.25
57 Kelley JM, Kraft-Todd G, Schapira L, *et al.* The influence of the patient-clinician relationship on healthcare outcomes: A systematic review and meta-analysis of randomized controlled trials. *PLoS One* 2014;**9**:e94207. doi:10.1371/journal.pone.0094207
58 Locher C, Meier S, Gaab J. Psychotherapy: A World of Meanings. *Front Psychol* 2019;:460. doi:10.3389/fpsyg.2019.00460
59 Lembo A, Kelley JM, Nee J, *et al.* Open-label placebo vs double-blind placebo for irritable bowel syndrome: a randomized clinical trial. *Pain* 2021;**Articles in Press**. doi:10.1097/j.pain.0000000000002234
60 Kirsch I. Response expectancy as a determinant of experience and behavior. *Am Psychol* 1985;**40**:1189–202. doi:10.1037/0003-066X.40.11.1189
61 Kirsch I. Conditioning, Expectancy, and the Placebo Effect: Comment on Stewart-Williams and Podd (2004). *Psychol Bull* 2004;**130**:341–3. doi:10.1037/0033-2909.130.2.341
62 Finniss DG, Kaptchuk TJ, Miller F, *et al.* Biological, clinical, and ethical advances of placebo effects. *Lancet* 2010;**375**:686–95. doi:10.1016/S0140-6736(09)61706-2
63 Petrie KJ, Rief W. Psychobiological Mechanisms of Placebo and Nocebo Effects: Pathways to Improve Treatments and Reduce Side Effects. *Annu Rev Psychol* 2019;**70**:599–625. doi:10.1146/annurev-psych-010418-102907
64 Wager TD, Atlas LY. The neuroscience of placebo effects: connecting context, learning and health. *Nat Rev Neurosci* 2015;**16**:403–18. doi:10.1038/nrn3976
65 Di Blasi Z, Crawford F, Bradley C, *et al.* Reactions to treatment debriefing among the participants of a placebo controlled trial. *BMC Health Serv Res* 2005;**5**:30. doi:10.1186/1472-6963-5-30
66 Stone DA, Kerr CE, Jacobson E, *et al.* Patient expectations in placebo-controlled randomized clinical trials. *J Eval Clin Pract* 2005;**11**:77–84. doi:10.1111/j.1365-2753.2004.00512.x
67 Smits RM, Veldhuijzen DS, Hartman TO, *et al.* Explaining placebo effects in an online survey study: Does 'Pavlov' ring a bell? *PLoS One* 2021;**16**:e0247103. doi:10.1371/journal.pone.0247103
68 Conboy L, Quilty MT, Kerr C, *et al.* A Qualitative Analysis of Adolescents' Experiences of Active and Sham Japanese-Style Acupuncture Protocols Administered in a Clinical Trial. *J Altern Complement Med* 2008;**14**:699–705. doi:10.1089/acm.2007.0718
69 Franklin J. Pascal's wager and the origins of decision theory: decision-making by real decision-makers. *Phil Archive* 2018;:27–44.
70 Ratnapalan M, Coghlan B, Tan M, *et al.* Placebos in primary care? a nominal group study explicating UK GP and patient views of six theoretically plausible models of placebo practice. *BMJ Open* 2020;**10**:e032524. doi:10.1136/bmjopen-2019-032524

For peer review only

71 Thielke SM, Turner JA, Shortreed SM, *et al.* Do Patient-perceived Pros and Cons of Opioids Predict Sustained Higher-Dose Use? *Clin J Pain* 2014;**30**:93–101. doi:10.1097/AJP.0b013e31828e361b

72 Geers AL, Wellman JA, Fowler SL, *et al.* Dispositional Optimism Predicts Placebo Analgesia. *J Pain* 2010;**11**:1165–71. doi:10.1016/j.jpain.2010.02.014

73 Evers AWM, Colloca L, Blease C, *et al.* What Should Clinicians Tell Patients about Placebo and Nocebo Effects? Practical Considerations Based on Expert Consensus. *Psychother Psychosom* 2021;**90**:49–56. doi:10.1159/000510738

For peer review only

Table 1
Sociodemographic characteristics of study participants

Group	N	Age (SD)	N (%) female	Family status	Highest educational level N	Employment level N	Credibility of the intervention N
OPR-	10	25.3 (6.63)	6 (60%)	Single: 10	Primary school: 0<br>Secondary school: 1<br>High school: 5<br>University: 4	Full time: 0<br>Part time: 3<br>None or student: 7	credible: 8<br>somewhat credible: 2<br>not credible: 0
DP	10	30.9 (14.65)	6 (60%)	Single: 9<br>Divorced: 1	Primary school: 1<br>Secondary school: 0<br>High school: 5<br>University: 4	Full time: 0<br>Part time: 5<br>None or student: 5	credible: 6<br>somewhat credible: 3<br>not credible: 1
OPR+	10	25.4 (4.86)	7 (70%)	Single: 10	Primary school: 0<br>Secondary school: 1<br>High school: 5<br>University: 4	Full time: 1<br>Part time: 5<br>None or student: 4	credible: 9<br>somewhat credible: 1<br>not credible: 0

Note. DP, deceptive placebo; OPR-, open-label placebo without rationale; OPR+, open-label placebo with rationale.

Table 2
Superordinate categories with corresponding main categories

Superordinate Categories	Main Categories
1. The placebo concept.	1.1. Descriptive
	1.2. Effectiveness
	1.3 Specific examples
2. Open-label placebo rationale.	2.1 First discussion point: "Placebos are powerful"
	2.2 Second discussion point: "The body automatically responds to placebos like Pavlov's dog"
	2.3 Third discussion point: "A positive attitude helps but is not necessary"
3. Experiences of taking part in an open-label placebo trial.	3.1. Reaction to (open-label) placebo
	3.2. Efficacy and effectiveness during the experiment

For peer review only

For peer review only

eAppendix 1. Topic Guide

1. Discourse

In general:

In your own words: what do you imagine a placebo to be?	
How do you think placebos work?	
What could be the cause/reason?	

Related to the experiment:

How did the experiment influence your understanding of placebos?	

2. Project

In general:

To what extent do placebos occur in your everyday life? (Active use of the placebo effect - e.g., in the form of home remedies)	
How significant are these effects and implications of a placebo for you?	

Related to the experiment:

Now if you had pain (e.g., heat pain, burning sensation) - would you use a cream described as "placebo"?	
If yes: Would you have taken this cream before experiencing this experiment?	

3. Selfhood

In general:

Do you think you respond particularly well/badly to placebos? Why?	
How much control do you think you have over the effect of a placebo?	

Related to the experiment:

What do you think - did the placebo cream have an effect on pain perception in this study? If so, what was the effect?	

4. Embodiment

In general:

For peer review only

Do you believe that a placebo can have an "effective" physical effect or is it merely an "imaginary" effect, i.e., a deception (of the senses)?	

Related to the experiment:

What was it like for you? Please describe the physical sensations of using the placebo cream.	

5. Sociality

In general:

To which people in your social environment would you recommend placebos? Why?	

Related to the experiment:

Will you tell people at home (with friends or relatives) about your experience with placebos that you had in the experiment?	
How will this make you feel towards the other person?	

6. Spatiality

In general:

Can you describe to me an environment or place where you feel, a placebo would work particularly well?	

Related to the experiment:

What impact did it have on you that you received the placebo in the context of an experiment and not in the "real world"?	

7. Temporality

In general:

How long do you think placebos last?	
When you think back - what significant memories with placebos come to mind? (e.g., comforting songs in childhood, remedies for a cold)	

Related to the experiment:

A glimpse into the future: Do you take anything away from the experiment and from this conversation with respect to placebos?	

For peer review only

eTable 1. Superordinate categories, main categories and categories

Superordinate categories, main categories and categories

Superordinate Categories	Main Categories	Categories	Frequencies		
			OPR-	OPR+	DP
1. The placebo concept.	1.1. Descriptive	Deception	4	6	10
		Study design component	1	0	1
		Inertness	7	9	6
		Substitute	2	0	0
	1.2. Effectiveness	Regularity of intake is necessary for effectiveness	2	1	2
		Sham effect	0	0	2
		Effect on body	9	3	7
		Psychological effect	3	1	1
		Paradox: Effect without active ingredient	6	5	1
		Effect-free	5	0	1
	1.3 Specific examples	Autosuggestion	1	1	1
		Spirituality	1	1	1
		Drug	0	4	2
		Interpersonal component	2	2	5
		Esotericism	1	0	1
		Deception	0	0	1
		Advertisement	0	0	1
		Complementary medicine	8	8	9
		Action	0	4	6
		Activity	2	3	6
		Food supplements	0	2	2
		Mindfulness	1	0	0
		Food products	9	8	11
2. Open-label placebo rationale.	2.1 First discussion point: "Placebos are powerful"	Less efficacy due to lack of deception	4	2	5
		OLP administration mitigates expectancy/belief	4	2	5
		OLPs work	0	1	0
		Surprise about OLPs effectiveness	0	2	0
		Effect of deceptive placebos lasts longer in contrast to OLPs	0	2	0
		Effect due to deceptive placebos, less effect due to OLPs	0	2	0
	2.2 Second discussion point: "The body automatically responds to placebos like Pavlov's dog"	Relationship	9	6	7
		Imagination	0	2	2
		Psychological factors	11	11	7
		Misattribution	1	0	0
		Mind-body-interaction	22	17	13
		Conditioning	4	11	2
		Ritual/action	3	5	1
		Openness/personality/attitudes	37	24	26
		Expectancy/belief/hope	28	27	30
	2.3 Third discussion	Depending on the experience	2	6	1
		If it helps and does no harm	0	0	1

For peer review only

	point: "A positive attitude helps but is not necessary"	If it's the only option	2	3	2
		It's worth a try	3	2	0
		Worth to see what happens	0	2	0
		As long as one believes that it helps	2	0	3
		Depending on the person	2	0	1
		Depending on the situation	0	1	0
		Depending on symptoms / disorder	4	4	3
3. Experiences of taking part in an open-label placebo trial.	3.1. Reaction to (open-label) placebo	When administered openly, skepticism arises as to whether an ingredient is not in the pill after all	2	1	0
		OLPs do not elicit wellbeing	0	1	0
		OLPs as something novel	1	6	0
		OLPs are incomprehensible/strange/crazy	5	1	1
		Openness towards OLPs	0	1	0
		Open administration is less popular	0	1	0
		OLP rationale is essential	0	3	0
		Placebo as something unknown	0	0	2
	3.2. Efficacy and effectiveness during the experiment	No efficacy due to the lack of deception	0	1	0
		Efficacy unlikely	1	3	0
		Efficacy only assumed posthoc	0	0	2
		Efficacy possible	4	1	4
		Efficacy unclear	1	3	0
		No objective efficacy (measures), but subjective efficacy (own sensation)	0	0	1
		Efficacy likely	1	0	4
		Positive experience due to cooling/experimental factors	4	3	4
		Positive experience on a psychological level	1	2	0
		No experienced efficacy in the experiment	9	9	5

Note. DP = deceptive placebo; OPR- = open-label placebo without rationale; OPR+ = open-label placebo with rationale.

For peer review only

Standards for Reporting Qualitative Research (SRQR)*

http://www.equator-network.org/reporting-guidelines/srqr/

Page/line no(s).

Title and abstract

**Title** - Concise description of the nature and topic of the study Identifying the study as qualitative or indicating the approach (e.g., ethnography, grounded theory) or data collection methods (e.g., interview, focus group) is recommended	p. 1 / line 1
**Abstract** - Summary of key elements of the study using the abstract format of the intended publication; typically includes background, purpose, methods, results, and conclusions	p. 2 / lines 26-44

Introduction

**Problem formulation** - Description and significance of the problem/phenomenon studied; review of relevant theory and empirical work; problem statement	p. 4-5 / lines 71-117
**Purpose or research question** - Purpose of the study and specific objectives or questions	p. 6 / lines 118-125

Methods

**Qualitative approach and research paradigm** - Qualitative approach (e.g., ethnography, grounded theory, case study, phenomenology, narrative research) and guiding theory if appropriate; identifying the research paradigm (e.g., postpositivist, constructivist/ interpretivist) is also recommended; rationale**	p. 7-8 / lines 157-162
**Researcher characteristics and reflexivity** - Researchers' characteristics that may influence the research, including personal attributes, qualifications/experience, relationship with participants, assumptions, and/or presuppositions; potential or actual interaction between researchers' characteristics and the research questions, approach, methods, results, and/or transferability	p. 7 / lines 153-156
**Context** - Setting/site and salient contextual factors; rationale**	
**Sampling strategy** - How and why research participants, documents, or events were selected; criteria for deciding when no further sampling was necessary (e.g., sampling saturation); rationale**	p. 6-7/ lines 133-139
**Ethical issues pertaining to human subjects** - Documentation of approval by an appropriate ethics review board and participant consent, or explanation for lack thereof; other confidentiality and data security issues	p. 6 / lines 131-132
**Data collection methods** - Types of data collected; details of data collection procedures including (as appropriate) start and stop dates of data collection and analysis, iterative process, triangulation of sources/methods, and modification of procedures in response to evolving study findings; rationale**	p. 7 / lines 142-145

For peer review only - http://bmjopen.bmj.com/site/about/guidelines.xhtml

**Data collection instruments and technologies** - Description of instruments (e.g., interview guides, questionnaires) and devices (e.g., audio recorders) used for data collection; if/how the instrument(s) changed over the course of the study	p. 7 / lines 145-149
**Units of study** - Number and relevant characteristics of participants, documents, or events included in the study; level of participation (could be reported in results)	p. 8-9 / lines 172-189
**Data processing** - Methods for processing data prior to and during analysis, including transcription, data entry, data management and security, verification of data integrity, data coding, and anonymization/de-identification of excerpts	p. 7 / lines 150-152
**Data analysis** - Process by which inferences, themes, etc., were identified and developed, including the researchers involved in data analysis; usually references a specific paradigm or approach; rationale**	p. 8 / lines 163-171
**Techniques to enhance trustworthiness** - Techniques to enhance trustworthiness and credibility of data analysis (e.g., member checking, audit trail, triangulation); rationale**	p. 8 / line 171

Results/findings

**Synthesis and interpretation** - Main findings (e.g., interpretations, inferences, and themes); might include development of a theory or model, or integration with prior research or theory	p. 9-18 / lines 191-391
**Links to empirical data** - Evidence (e.g., quotes, field notes, text excerpts, photographs) to substantiate analytic findings	p. 18 / lines 402-404

Discussion

**Integration with prior work, implications, transferability, and contribution(s) to the field** - Short summary of main findings; explanation of how findings and conclusions connect to, support, elaborate on, or challenge conclusions of earlier scholarship; discussion of scope of application/generalizability; identification of unique contribution(s) to scholarship in a discipline or field	p. 18-23 / lines 392-508
**Limitations** - Trustworthiness and limitations of findings	p. 23 / lines 509-525

Other

**Conflicts of interest** - Potential sources of influence or perceived influence on study conduct and conclusions; how these were managed	p. 3 / line 65
**Funding** - Sources of funding and other support; role of funders in data collection, interpretation, and reporting	p. 3 / lines 63-64

*The authors created the SRQR by searching the literature to identify guidelines, reporting standards, and critical appraisal criteria for qualitative research; reviewing the reference lists of retrieved sources; and contacting experts to gain feedback. The SRQR aims to improve the transparency of all aspects of qualitative research by providing clear standards for reporting qualitative research.

For peer review only

**The rationale should briefly discuss the justification for choosing that theory, approach, method, or technique rather than other options available, the assumptions and limitations implicit in those choices, and how those choices influence study conclusions and transferability. As appropriate, the rationale for several items might be discussed together.

Reference:

O'Brien BC, Harris IB, Beckman TJ, Reed DA, Cook DA. **Standards for reporting qualitative research: a synthesis of recommendations.** *Academic Medicine*, Vol. 89, No. 9 / Sept 2014 DOI: 10.1097/ACM.0000000000000388

For peer review only

For peer review only - http://bmjopen.bmj.com/site/about/guidelines.xhtml

BMJ Open

Lay perspectives of the open-label placebo rationale: A qualitative study of participants in an experimental trial

Journal:	*BMJ Open*
Manuscript ID	bmjopen-2021-053346.R1
Article Type:	Original research
Date Submitted by the Author:	14-Jul-2021
Complete List of Authors:	Locher, Cosima; University Hospital Zurich, Department of Consultation-Liaison Psychiatry and Psychosomatics; University of Basel, Faculty of Psychology<br>Buergler, Sarah; University of Basel<br>Frey Nascimento, Antje; University of Basel, Faculty of Psychology<br>Kost, Linda; University of Basel<br>Blease, Charlotte; Harvard Medical School, General Medicine and Primary Care<br>Gaab, Jens; University of Basel
**Primary Subject Heading**:	Qualitative research
Secondary Subject Heading:	Ethics, Health services research, Patient-centred medicine
Keywords:	QUALITATIVE RESEARCH, Quality in health care < HEALTH SERVICES ADMINISTRATION & MANAGEMENT, MENTAL HEALTH, PAIN MANAGEMENT, PRIMARY CARE

SCHOLARONE™
Manuscripts

I, the Submitting Author has the right to grant and does grant on behalf of all authors of the Work (as defined in the below author licence), an exclusive licence and/or a non-exclusive licence for contributions from authors who are: i) UK Crown employees; ii) where BMJ has agreed a CC-BY licence shall apply, and/or iii) in accordance with the terms applicable for US Federal Government officers or employees acting as part of their official duties; on a worldwide, perpetual, irrevocable, royalty-free basis to BMJ Publishing Group Ltd ("BMJ") its licensees and where the relevant Journal is co-owned by BMJ to the co-owners of the Journal, to publish the Work in this journal and any other BMJ products and to exploit all rights, as set out in our licence.

The Submitting Author accepts and understands that any supply made under these terms is made by BMJ to the Submitting Author unless you are acting as an employee on behalf of your employer or a postgraduate student of an affiliated institution which is paying any applicable article publishing charge ("APC") for Open Access articles. Where the Submitting Author wishes to make the Work available on an Open Access basis (and intends to pay the relevant APC), the terms of reuse of such Open Access shall be governed by a Creative Commons licence – details of these licences and which Creative Commons licence will apply to this Work are set out in our licence referred to above.

Other than as permitted in any relevant BMJ Author's Self Archiving Policies, I confirm this Work has not been accepted for publication elsewhere, is not being considered for publication elsewhere and does not duplicate material already published. I confirm all authors consent to publication of this Work and authorise the granting of this licence.

For peer review only

Lay perspectives of the open-label placebo rationale: A qualitative study of participants in an experimental trial

Cosima Locher[1,2,3], Sarah Buergler[3], Antje Frey Nascimento[3], Linda Kost[3], Charlotte Blease[4], & Jens Gaab[3]

[1] Department of Consultation-Liaison Psychiatry and Psychosomatic Medicine, University Hospital Zurich, Switzerland
[2] Faculty of Health, University of Plymouth, Plymouth, UK
[3] Division of Clinical Psychology and Psychotherapy, Faculty of Psychology, University of Basel, Switzerland
[4] General Medicine and Primary Care, Beth Israel Deaconess Medical Center, Harvard Medical School, Boston, USA

Corresponding author: Cosima Locher, PhD, Department of Consultation-Liaison Psychiatry and Psychosomatic Medicine, University Hospital Zurich, Switzerland. Email: cosimaantoinette.locher@uzh.ch Phone: 0041 79 461 32 54

Keywords: open-label placebo, rationale, mechanisms, qualitative, thematic analysis

Short title: Open-label placebo rationale revisited

Word count: 5995

For peer review only

Abstract

Objectives: To analyze participants' concepts about the open-label placebo (OLP) effect; to explore their views about the discussion points that are applied in conventional OLP trials; and to examine their experiences of taking part in an OLP trial.

Design: A qualitative study using thematic analysis of semi-structured interviews that was nested within a randomized controlled trial investigating experimental OLP analgesia.

Participants: Thirty healthy adults who took part in the randomized controlled trial.

Results: Participants mostly conceptualized placebo as something that is inert and requires deception in order to be effective. Interviewees used a broad definition of placebos, going beyond a conventional notion of sugar pills. In contrast to the conventional OLP rationale, participants seldom emphasized classical conditioning as a mechanism of placebo effects, stressing a variety of other well-established components through which placebos might be therapeutic. Whereas the conventional OLP disclosures state that "a positive attitude helps but is not necessary", participants in our study applied other attitudes, such as "it's worth a try". When asked about their experiences during the trial, the majority emphasized that the concept of OLP

For peer review only

was completely novel to them. Participants were rather skeptical about the efficacy of the intervention.

Conclusion: Integrating lay perspectives into the scientific rationale of OLP treatments might enhance the plausibility and credibility of the rationale in ethical treatments.

For peer review only

For peer review only - http://bmjopen.bmj.com/site/about/guidelines.xhtml

Strengths and limitations of this study

- Despite the increasing number of OLP trials, this is, to the best of our knowledge, the first qualitative study that examined participants' attitudes about OLP
- The semi-structured interviews generated rich, contextual data
- The sample consisted of young university students which may limit generalizability

Additional statements

Twitter: @the_pain_net

Acknowledgments: The authors would like to thank Xenia Knüsel and Sebastian Hasler for their assistance with the data transcription. They would also like to thank Berfin Bakis for her help with the translation of the quotations. We also thank Mareike Rytz for her help with the references.

Contributors: CL, CB, and JG: study concept and design; CL: conducted all interviews and drafted the manuscript; CL, SB, AF, and LK: coded all interviews; CB, JG, SB, AF, and LK: contributed to critical advice and revisions of the manuscript. All authors read and approved the final manuscript.

Funding statement: CL received funding from the Swiss National Science Foundation (SNSF): P4P4PS_194536.

Competing interests statement: The authors have no conflicts of interest to disclose.

Data availability statement: Anonymized transcripts of the qualitative interviews are stored at the *Harvard Dataverse* data repository: https://doi.org/10.7910/DVN/ET5PVQ

ORCID ID: Cosima Locher: https://orcid.org/0000-0002-9660-0590

Patient and public involvement: Patients and the public were not involved in the design of the study; however, the study specifically focuses on participant perceptions.

For peer review only

Introduction

There is evidence that placebos exhibit substantial effects with promising clinical potential in various mental and physical complaints [1]. Placebos in randomized controlled trials usually refer to dummy medications (e.g., sugar pills) which in theory are indistinguishable from the treatment under investigation and which serve as methodological tools to screen out confounders that are associated with clinical trials (e.g., spontaneous remission, regression to the mean) [2]. In contrast, placebos in clinical practice are interventions without known specific effects on the treated condition but used with the goal of achieving positive outcomes by eliciting placebo effects (e.g., through treatment expectations) [3]. However, the potential of placebo effects in clinical practice is impeded as its administration requires deception. Some health researchers have argued that deceptive placebos may sometimes be ethical in the interests of therapeutic gain – for example, where no other treatment options are available, or on the grounds that the deception is trivial and does not involve a major threat to patient autonomy [4,5]. However, most ethicists argue that deception in clinical context is unjustified and violates duties to be honest with patients [6,7]. In addition, deception may risk derailing trust in clinicians, leading to possible harms including disengagement with healthcare [8,9].

Here, openly prescribed placebos offer the possibility to harness placebo effects in a transparent and thus ethical way [10]. The OLP approach has been examined in various physical and mental disorders, e.g., chronic low back pain [11,12], irritable bowel syndrome [13], allergic rhinitis [14–16], ADHD [17,18], and fatigue among cancer survivors [19,20]. So far, two meta-analysis examining OLPs revealed that patients in an OLP condition exhibit significantly greater improvement than those in a control group [21,22]. There is evidence that OLPs may have significant effects on sub-clinical conditions, e.g., menopausal hot flushes [23], and test anxiety

For peer review only

[24,25], as well as on well-being, e.g., sleep quality [26], and wound healing [27]. OLP has also been investigated in experimental settings such as experimentally induced pain, and allergic reactions [28–31].

All of these studies clearly inform participants that the intervention consists of "honestly described placebo pills" [32], applying a plausible and positive rationale. A standard approach has emerged with these studies, whereby disclosure about the rationale conveys four discussion points, which are based on both study findings and clinical considerations [13,32]: (1) *Placebos are powerful*. The major aim of this statement is to remove the stigma of placebo effects and to openly inform participants about the wide array of study findings that report powerful placebo effects in double-blind randomized controlled trials (RCTs) [32]. (2) *The body can automatically respond to taking placebo pills like Pavlov's dogs who salivated when they heard a bell*. The second discussion point emphasizes classical conditioning as a major mechanism of how placebos might elicit therapeutic effects [33,34]. (3) *A positive attitude helps but is not necessary*. The aim of the third key point may be designed to take participants' skepticism seriously and to emphasize that they do not have to believe in the treatment for it to work [32]. (4) *Taking the pills faithfully is critical*. The final discussion point is related to study findings from RCTs that reveal that adherence is crucial for a (medical) treatment to work [35,36]. In this paper, we describe OLP studies that focus on these discussion points as "conventional OLP" disclosures.

From an ethical point of view, transparency in the OLP rationale is crucial in order to respect participants' autonomy [8,37]. So far, two qualitative investigations have examined what participants experience when they are informed that they were assigned to the placebo arm in a placebo-controlled clinical trial [38,39]. These studies reveal that participants hesitate and doubt

For peer review only

whether the placebo treatment has a potential to heal, yet many patients also spontaneously express hope [38]. Although informative, these findings are not transferable to the field of OLP, where participants know that they will receive a placebo. There is only one pilot study that examined participants' perspectives on the OLP treatment [40]. In that study, 10 participants were asked about their definition of placebo, whether the OLP rationale was clear to them, and whether anything about the study was misleading. None of the questions, however, solicited feedback on attitudes about OLP.

Therefore, this qualitative study set out to investigate participants' attitudes about (open-label) placebo. The study was nested within a RCT that investigated OLP analgesia in healthy participants. The aims of the study were threefold: First, to analyze participants' concepts about the (open-label) placebo effect. Second, to compare participants' views on OLP with discussion points that are applied in conventional OLP trials. Third, to summarize participants' experiences of taking part in an OLP trial. For all our aims, we strove to compare participants' statements of the three intervention groups of the quantitative study, i.e., open-label without rationale (OPR-), open-label placebo with rationale (OPR+), and deceptive placebo (DP) groups.

Methods

Study design

This is a mixed methods qualitative study embedded within a RCT investigating the OLP treatment in healthy participants [30]. The study applied an experimental heat pain paradigm, comparing four groups, i.e., no treatment (NT), OPR-, OPR+, and DP. The rationale that was

For peer review only

provided to the three groups can be found in the supplement (see eAppendix 1). In the quantitative part that has been published elsewhere [30], we found that the OPR+ group exhibited a significant reduction of subjective heat pain ratings that did not differ from the DP group. For the qualitative part of the study, semi-structured interviews were scheduled at the end of each participant's treatment.

Study participants

Thirty participants from the intervention groups (i.e., OPR-, OPR+, and DP) out of the total sample of 160 were randomly selected to take part in the nested qualitative study. Participants were healthy adults from the general population recruited through advertisements. None of the participants were Psychology or Medicine students due to potential prior knowledge regarding placebo mechanisms and effects. Interviewing participants from the different intervention groups allowed us to examine attitudes towards the OLP approach in dependence on previous experimental experiences. Table 1 shows participants' basic demographic information. Recruitment started in January 2016 and lasted until July 2016. Written informed consent was obtained from each participant.

Interview procedure

The interviews were conducted by a trained interviewer (CL) applying a semi-standardized interview guide. All participants have been interviewed about their attitudes towards the (open-label) placebo effect. In order to ensure that participants who did not receive the OLP treatment (i.e., OPR- and DP groups) are not primed by the existing open-label placebo rationale, we aimed to explore their attitudes towards the placebo effect with a bottom-up approach. The interview covered the following components: (a) identity of the placebo effect, (b) possible causes of the placebo effect, (c) opinions about short- and long-term consequences of the placebo

For peer review only

effect, (d) the perceived control over the placebo effect and (e) the timeline of the placebo effect. A broad interview guide was prepared comprising 21 questions as a tool to obtain information (see eAppendix 2). Interviews lasted between 21 and 66 minutes. The interviews were audio-taped and verbatim transcripts were written. The listed quotations have been translated from German to English.

The first author who conducted the interviews (CL) completed her PhD in 2017. In her research, she focuses on the effects, mechanisms, and routes of administration of (open-label) placebo. For that, she applies different methodological approaches, encompassing qualitative studies, experimental trials, clinical investigations, and (network) meta-analyses.

Qualitative Analysis

For data analysis, MAXQDA 2020 (VERBI Software, 2019) was used [41]. We followed an inductive-deductive hybrid approach [42] and incorporated primarily an inductive data-driven approach to allow the examination of core themes for a phenomenon with limited existing theory or research literature [43–45], complemented by a deductive ideation of the theoretical points of the OLP treatment rationale [46]. With the aim to describe common patterns across the interviews, the following six steps were performed [47]: First, the comment transcripts were read 3-4 times by four coders (SB, LK, AF, and CL) in order to achieve familiarization with the data. Second, the two main coders (SB and LK) identified brief descriptive labels ('codes') that represented different aspects of interviewees' descriptions. More than a single code was applied if quotations had multiple meanings. Third, the four coders (SB, LK, AF, and CL) examined the codes and sorted them into different themes with shared meaning units. Fourth, the themes were reviewed, i.e., themes that were not supported by the data were modified by the coders. Fifth, the

For peer review only - http://bmjopen.bmj.com/site/about/guidelines.xhtml

coders developed a detailed analysis of each theme by defining final names. Finally, all authors took part in authoring the manuscript. We sought analytical rigor by involving multiple coders.

Results

Participants

Participants had a mean age of 27.2 (SD 9.73) years, and 63% of the participants were female (see Table 1). The three groups were comparable regarding age ($F(2,27) = 1.09$, $P = 0.35$), sex ($X^2(2) = 0.29$, $P = 0.866$), family status ($X^2(4) = 3.61$, $P = 0.462$), educational level ($X^2(6) = 3.00$, $P = 0.809$), and employment level ($X^2(4) = 3.49$, $P = 0.479$). Quantitative results were reported elsewhere [30,48]. The majority of participants assigned to the OPR- and OPR+ groups believed they were receiving a placebo cream (17/20). Most participants in the deceptive placebo group also believed they were receiving the analgesic cream (6/10).

Overview

In the 30 interviews, 667 text passages of 30’379 words were identified and summarized into 65 categories. These categories were subsumed in 8 main categories, which were classified in three superordinate categories: (1) the placebo concept; (2) open-label placebo rationale; and (3) experiences of taking part in an open-label placebo trial (see Table 2). An overview of all superordinate categories, main categories and categories is provided in the supplement, also revealing the number of quotations for each category per group (eTable 1). In the following, we present the four superordinate categories and main categories. For the complete list of categories please see Table 2.

1. Placebo concept

1.1 Descriptive

For peer review only

The vast majority of participants agreed on the definition of placebo as something that is characterized by its inertness and its deceptive administration. This assumption was independent of group allocation; for example:

- *"To me, a placebo is a drug without actual ingredients"* (DP; participant 42)

Likewise, many participants emphasized that deception is a prerequisite for placebos to work. Notably, examples not only included the classical placebo pill, yet also deceptive aspects in politics and advertisements that lead to a change of belief or behavior; for example:

- *"Well, in term of deception - so when you use the term deception, I don't know, in politics that happens; I often feel like you can achieve something with deceptive means"* (OPR-; participant 89)

A few comments linked the placebo concept to the design and conductance of clinical studies, such as randomized controlled trials; for example:

- *"Well, I've heard that quite often now, that, I don't know, experiments or other studies were conducted where people were given placebos"* (OPR-; participant 56)

Notably, two participants from the OPR- group expressed the view that placebos are like a copy or a substitute of a verum intervention, which is, however, less powerful:

- *"Yes, some kind of substitute, like - just like this clone phenomenon, that is kind of like a copy but not quite the same, and also not as good <<laughs>>"* (OPR-; participant 135)

1.2. Effectiveness

Interviewees expressed a variety of perspectives about the effectiveness of placebos. Especially participants who were allocated to the OPR+ and OPR- groups emphasized the paradox that placebos are inert, yet reveal positive effects; for example:

For peer review only

- *"When I think of a 'drug' it's a drug that doesn't have any medical ingredients; it's like neutral actually yet triggers an effect afterwards"* (OPR+; participant 141)

Similarly, many participants indicated general agreement that the intake of placebos has an effect on the body. This was independent of group allocation; for example:

- *"[Mhm] (.) more than an imagination actually, with actual physical effects"* (OPR+; participant 109)

In opposition to this view, other interviewees neglected that placebos can have a physical effect. Some interviewees stressed that placebos are genuinely free of effect, also using terms like "sham" and "fake". Notably, except one participant, all statements stemmed from participants assigned to the OPR- or DP groups; for example:

- *"No no no, it's really only controlled by the mind in our head, so one should not be able to measure anything purely physically"*(OPR-; participant 89)

1.3. Specific examples

Building on this theme, the vast majority of interviewees offered a variety of examples of, what they considered to be, placebos. Examples went beyond the classical pill encompassing autosuggestion, esotericism, complementary medicine, food supplements, advertisement, mindfulness, and food. Notably, patients allocated to the OPR+ group gave more context to these statements, explaining why they think that these kinds of placebos might work:

- *"Yes, it probably is the case that sometimes, when I notice that I'm getting a cold or have a cold, I just take and eat ginger, and I'm convinced that it works every single time. In this case I also feel like it works <<laughs>>; and I can certainly imagine that it just…that these rituals also have an effect, yes"* (OPR+; participant 157)

For peer review only

More broadly, and often related to daily life experiences, interviewees also mentioned that a placebo can be a general act, an activity, or something that happens in the interpersonal contact; for example:

- *"The sauna, for example, I do it regularly as soon as winter starts. And was never sick ever since"* (OPR-; participant 74)
- *"I always watch TV in order to fall asleep. I fall asleep faster when I do that than when I don't. A placebo is also like a ritual, yes"* (OPR+; participant 146)

2. Open-label placebo rationale

All interviewees stressed components that can be linked to the established OLP rationale. Whereas some aspects were in line with the discussion points of the rationale, others were complementary or even contradictory:

2.1 First discussion point

The majority of interviewees concretized the anticipated effect of an OLP treatment, which is related to the first discussion point of the OLP rationale (i.e., *placebos are powerful*). Participants agreed that an OLP treatment is less effective and/or has shorter lived effects than the deceptive placebo treatment; for example:

- *"But I feel like if you know that it's a placebo, it may not be as long-lasting than if you don't"* (OPR+; participant 146)

More precisely, interviewees stated that key mechanisms such as positive expectations and belief systems are reduced in the OLP treatment. It was also argued that the lack of deception is associated with a significant reduction of placebo efficacy. Most concerns were expressed from

For peer review only

participants assigned to the OPR- and DP group with the exception of two people from the OPR+ group; for example:

- *"[Hmm] well, I believe the whole belief system collapses a little bit; I mean when you have a very medical name and an appealing packaging, it will work even if there's a placebo pill inside"* (OPR-; participant 74)
- *"For me a placebo is something that is really connected to a strong belief, so I have a feeling that it only works, or especially works, when for example one doesn't know that it is a placebo"* (OPR+; participant 141)

Some interviewees from the OPR+ group, however, believed that an OLP treatment can be powerful; for example:

- *"Yes, even if it's just a placebo, uhm, simply from the studies that have been conducted so far, one knows that it can work; and already that, that statement has had an effect on me and I thought 'well okay so even if it's open it still works a little bit' so"* (OPR+; participant 35)

2.2 Second discussion point

Interviewees listed a big variety of mechanisms that make the OLP treatment work. In line with the second discussion point of the established OLP rationale (i.e., *the body can automatically respond to taking placebo pills like Pavlov's dogs who salivated when they heard a bell)*, some interviewees emphasized classical conditioning as a key mechanism for placebos to be effective. Notably, only participants from the OPR+ group explicitly referred to the term 'conditioning' whereas others talked about the impact of experiences and unconscious processes; for example:

For peer review only

- *"Yes, because we're used to it, just like for example when I saw the drug and had the package in my hand, yes for me it's association so it's a drug, only drugs look like that"* (OPR+; participant 35)

Notably, however, comments that could be linked to 'conditioning' represented only a minority of interviewees' statements. The vast majority highlighted the impact of personality traits such as openness and a positive attitude. This was independent of group allocation; for example:

- *"I think I'm pretty open-minded regarding new things, maybe that has something to do with it, too"* (OPR+; participant 141)

Besides people's personality components and general attitudes, interviewees also stressed personal mindsets that are directed towards the future, encompassing positive expectancies, beliefs and hope; for example:

- *"[Hmm] or from the expectations one might have; I can imagine that simply through the doctor administering something, like a preparation, people will generate certain expectations"* (OPR+; participant 109)

More broadly, participants acknowledged the mind-body connection and psychological components in general; for example:

- *"Well, it's not that something just happens by mind power, but something in my body changes through mind power. So, there's definitely something happening on a molecular basis, that's clear" (*DP; participant 154)

Besides components that are linked to the individual patient, other comments underlined the impact of a trust-based patient-provider relationship. Again, this was independent of group allocation; for example:

For peer review only

For peer review only - http://bmjopen.bmj.com/site/about/guidelines.xhtml

- *"So, I took it when I was a kid because my doctor recommended it to me, and I mean as a kid you trust doctors 100% anyway, right? <<laughs>>and I've always felt very comfortable in that practice, that's also very important. And the investigator [of the study] was very nice and always explained exactly what she was doing, I think that there's a big connection" (*OPR-; participant 99)
- *"Absolutely, so I had one [an investigator] who had long conversations with me, and consequently one is- you're more easily inclined to engage in something when you feel like you're in good hands and he really took his time too so, yeah" (*DP; participant 48)

Some interviewees highlighted other placebo mechanisms such as the power of imagination and the ritual. Notably, one participant also emphasized that a response to receiving a placebo might also encompass the natural course of the disease:

- *"So, in a first step someone takes a placebo, and by pure chance their headache fades away. Well not in that exact moment, but after a couple of minutes, on its own, right? [interviewer: like a natural course?] Exactly. So, the pain comes and goes without taking any painkillers"* (OPR-; participant 39)

2.3 Third discussion point

The third discussion point of the original OLP rationale (i.e., *a positive attitude helps but is not necessary*) has the general aim to take participants' skepticism seriously and to operate as a kind of relativization. Crucially, none of the participants spontaneously expressed such a thought. However, they referred to other aspects of relativization that would encourage them to take an OLP, such as the idea of "nothing to lose" and "it's worth to give it a try"; for example:

For peer review only

- *"I'd probably do it in a way where I would give the cream a chance and say, 'it doesn't do me any harm, just give it a try.'"* (OPR-; participant 89)
- *"You actually can't lose anything at all, that's another aspect of it, I think. It's definitely worth a try"* (OPR+; participant 109)
- *"Well, if it helps and doesn't harm my body in any way, then that's great"* (DP; participant 138)

Others agreed that the OLP treatment would be an option for them *if* there would be no alternative; for example:

- *"Good question, I think it depends on the situation, like how alarming it is. If I wouldn't have anything but the placebo cream, I'd probably use it <<laughs>>"* (OPR-; participant 135)

On a broader scale, interviewees speculated that the readiness to take an OLP treatment also depends on the context and the respective disorders; for example:

- *"As I said, you need to differentiate of course, as there are different forms of diseases; when I talk about the common cold or cancer therapy, those are clearly two different things, and of course you have to differentiate"* (DP; participant 138)

3. Experiences of taking part in an open-label placebo trial

3.1. Reaction to (open-label) placebo

The vast majority of participants assigned to the OPR+ group emphasized that the OLP approach was completely novel to them; for example:

For peer review only

- *"Um so I didn't know that there's research - well that there's research showing that there are effects when you know that it's a placebo and it still has an effect"* (OPR+; participant 146)

Furthermore, patients that were allocated to the OPR+ group agreed that the OLP treatment was only plausible because of the rationale that was provided during the experiment; for example:

- *"How should I say, that there's still the release of some substances; now I'm not very familiar with this <<laughs>>. But uhm I think when she [the investigator] told me about it, that it's like conditioned, that seemed plausible to me"* (OPR+; participant 141)

Opposing to this, a dominant theme in the OPR- group was the conviction that the OLP treatment is a little strange, crazy and incomprehensible; for example:

- *"But that's not negative at all, if anything I think it's a funny farce << laughs >>"* (OPR-; participant 135)
- *"It is very peculiar"* (OPR-; participant 74)

Interestingly, three participants (i.e., two from the OPR- group and one from the OPR+ group) stated that they were speculating during the experiment whether they really received a placebo; for example:

- *"You might also think 'ah maybe it's not a placebo, maybe it's real cream and they won't tell me just to see- maybe to see how I react when I don't know that it contains active ingredients, and then has an effect anyway'"* (OPR-; participant 57)

3.2. Efficacy and effectiveness during the experiment

Finally, interviewees also made estimates about the efficacy of placebos in the experiment. The majority of participants of the OPR+ and OPR- groups suggested that the intervention was

For peer review only

probably not effective. Some participants from the DP group shared this impression; for example:

- *"No, I don't think so << both laugh >>, I don't think it worked, I even checked: it [the skin] was red right away, so yeah, I don't think it really worked"* (OPR-; participant 57)
- *"To be honest, I don't think it made a difference"* (OPR+; participant 34)
- *"I did not notice a difference"* (DP; participant 75)

On the contrary, some participants of the DP group reported that the intervention had an effect on their pain levels; likewise, some interviewees from the OPR+ and OPR- groups suggested that the intervention had an effect on their mind; for example:

- *"I really think, psychologically, because I um was told that with open placebos, well, the trials where people knew it's a placebo, that it worked there. I think it was through that statement actually, yes"* (OPR+; participant 35)

Some participants were also unsure about whether the intervention had an effect or not; for example:

- *"Well, I was wondering if it had an effect on my pain threshold, I don't know the results. And um, I tried to convince myself that it [the placebo] doesn't have an effect, I should be on the same threshold level [in the second round], yet there was this little thought 'it could still be effective'"* (OPR-; participant 89)

Notably, some interviewees reported that experimental components such as the cooling of the cream or the patch position had an influence on their pain experience; for example:

- *"But maybe I just thought um, just the cream, not the active ingredient is having an effect, just the layer << laughs >> between the plate and the skin"* (OPR-; participant 89)

For peer review only

Discussion

This qualitative study set out to evaluate participants' concepts about the (open-label) placebo effect, to compare participants' attitudes towards (open-label) placebos with the three discussion points of the conventional OLP disclosures, and to summarize participants' experiences during the experimental OLP trial. The present study is one of the first qualitative studies that is nested within an OLP trial.

We found that the vast majority of participants describe placebos as an intervention that is medically inert with respect to its ingredients for treating symptoms but efficacious and powerful. This is also in line with a pilot study that examined ten patients who received OLPs as an adjunctive treatment to opioids for acute pain. There, the majority of participants described placebos as something that is inactive [40]. To define a placebo as an inert substance that has no effect on human physiology is common, yet questionable. Several researchers argued that no substance is inert if "inert" is defined as something that has no physiological effect [49–51]. Inertness may also be associated with something that is neutral or unspecific. Placebos, however, reveal specific effects and mechanisms and can thus be conceptualized as a specific agent [52]. These contrasting views raise the question of whether it would be helpful to inform participants in OLP trials that placebos are specific by their nature. Another component that has been traditionally linked with the placebo term and that has been raised by the participants of our study is the assumption that placebos need deception in order to work. Particularly in clinical practice, however, it is clearly unethical to deceive patients by prescribing placebos as deception violates the principle of respect for patient autonomy [53]. In contrast, our results reveal that the long-time association of placebo and deception may be common in the general population. This implies that the information given in OLP trials, i.e., that placebos can also work when delivered

For peer review only

openly, is a crucial component of the OLP treatment. Interviewees described several kinds of placebos, going beyond the classical sugar pill. Notably, the spectrum of examples encompassed several aspects that have been studied in placebo research. The power of autosuggestion, for example, has been explained by a change of mind-set and a reappraisal of a situation [54]. Likewise, the practice of mindfulness has been supposed to modify mind-sets and has shown to be related to positive health outcomes [55]. Interviewees also related supportive interpersonal aspects to placebo effects, which is in line with research indicating that the alliance between the treatment provider and treatment recipient is related to the effects of the respective treatment, even if this treatment is placebo [56–59]. Finally, as outlined by some participants, religiosity and spirituality are related to hope, positive thinking, and meaning-making, all mechanisms that have been suggested to operate in placebos [e.g., 60]. Taken together, interviewees spontaneously outlined several kinds of placebos that have been the subject of multiple research approaches; this is in line with recent attempts to generalize placebos and to study them outside the medical context [e.g., 8].

The statements given by the interviewees were further compared with the conventional OLP rationale. Interviewees did not explicitly engage with the first discussion point (i.e., *placebos are powerful*) despite interviewer attempts in the OPR+ group, i.e., most made no statements in relation to the effectiveness of placebos, yet agreed that *openly* prescribed placebos might be less effective than deceptive placebos. This is in contrast to some first indications showing that openly described placebos work as well as deceptive placebos [30,61]. In order to inform patients that honestly prescribed placebos are also powerful, newer OLP trials started to outline the findings from the first controlled OLP trial [13], usually showing a video sequence of

For peer review only

an American NBC news report of the respective study [e.g., 11]. Our findings indicate that it is of utmost importance to inform participants about the promising evidence of OLP trials.

In relation to the second discussion point (i.e., *the body can automatically respond to taking placebo pills like Pavlov's dogs*), which clearly refers to one of the most important mechanisms of placebo effects (i.e., classical conditioning), quotations from our participants were surprising. We found that only one participant from the OPR+ group mentioned conditioning. Evidence in the field of conditioning also suggests that behavior cannot only be triggered by verbal information, yet also by nonconscious stimuli, a differentiation that was not mentioned by our participants. Besides classical conditioning, the second most dominant conceptual model to explain placebo effects is expectancy [62,63], a mechanism that was frequently stressed by our interviewees, also in relation to general beliefs and habits. A third mechanism that has received considerable attention and has shown to induce placebo effects is the patient-provider relationship [3], a component that was also emphasized by many of our interviewees, especially in relation to trust. Notably, participants of our study stressed a wide array of other mechanisms through which placebos work, including general attitudes, beliefs, hope, the ritual, and the power of imagination. All these factors are established mechanisms in placebo research [for an overview, see e.g., 64], usually subsumed under external and internal elements of the treatment context [65]. Another psychological component that received less attention from placebo researchers and that was frequently addressed by our participants is hope. This is in line with other qualitative placebo (yet not openly prescribed placebo) studies reporting that patients spontaneously expressed hope, which they usually explicitly distinguish from expectation [38,66,67]. Whereas expectancy is linked to cognitive states and a prediction of the future, hope is not so much a prediction but rather more an emotional state [38]. Taken together,

For peer review only

our findings reveal that participants of an OLP trial intuitively list a wide array of established placebo mechanisms. This raises the question why classical conditioning is *exclusively* stressed by the conventional OLP disclosures. A recent survey study that examined participants' preferences of placebo explanations found that other mechanisms than classical conditioning (i.e., positive expectations and brain mechanisms) were rated most preferable [68]. Mechanisms that have been shown to be relevant for the efficacy of placebos cannot be transferred to the field of openly prescribed placebos. It has been argued that patient expectations about the efficacy of a treatment substantially differ between conventional and open-label placebo approaches [29]. Thus, it might be worth considering informing participants in OLP trials about the variety of mechanisms that operate in *openly* prescribed placebos. This will ensure that the provided information is based on newest evidence.

Finally, the third discussion point of the conventional OLP rationale (i.e., *a positive attitude helps but is not necessary*) aims to reduce skepticism and works as a kind of relativization. Although interviewees of our study expressed different strategies to reinforce realistic (and not too high) expectations, none of them used the reasoning that *a positive attitude helps but is not necessary* or comparable relativization strategies. Interviewees argued that "it's worth a try"; "if it helps and does no harm", or "worth to see what happens". These viewpoints are not new: patients in randomized, sham-controlled trials who do not know whether they are assigned to the verum or the sham condition often argue that it is worth giving it a try [38,39,69]. The underlying heuristic appears to be: "I lose little if the placebo does not work, but gain a lot if it does work" [70]. Notably, some participants also outlined that they would take a placebo "if it's the only option". This is related to the notion that their openness would "depend on the symptoms or disorder", a finding that is in line with a previous qualitative study, applying a

For peer review only - http://bmjopen.bmj.com/site/about/guidelines.xhtml

scenario to explain OLP to participants [71]. Patients with chronic primary pain, for example, often describe that different treatment approaches have turned out to be unsatisfactory, leading to a perceived lack of alternatives and a state of demoralization [72], which might lead to an openness towards OLPs. Therefore, the idea of informing participants that *a positive attitude helps but is not necessary* might be problematic: not only do participants use other strategies to reduce skepticism, they are also convinced that positive attitudes are an important mechanism for placebos to work – a sentiment which is supported by current scientific evidence [73].

Finally, we were interested in participants' experiences in an experimental OLP trial. We found that for the vast majority of interviewees, the OLP approach was completely novel. Most interviewees had the impression that the treatment was non-effective. Slightly more participants from the DP group stated that the efficacy of the intervention was possible or even likely. Especially participants assigned to the OPR+ group mentioned that the efficacy of the intervention was "unclear". Interestingly, slightly more participants from the OPR- group stated that the efficacy of the intervention is "possible" when compared to the OPR+ group. These rather more skeptical and reserved expressions related to the efficacy of the intervention, that we found independent of group assignment, are in contrast to results of the original RCT. There, groups with a rationale (OPR+ and DP) reported significantly lower heat pain intensity and unpleasantness ratings than the OPR- group [30]. Beyond the presumed efficacy of the intervention, there were some group-dependent reactions to the concept of OLP. Whereas participants from the OPR+ group stressed that the plausible rationale was probably the most important component of the treatment, participants from the OPR- group said that the intervention was "strange, crazy, and incomprehensible". The differing views of these two

For peer review only

groups underscore the importance of a plausible explanation of the concept of OLPs to counter possible skepticism.

Strengths and limitations

The present qualitative study has a number of limitations. The OLP trial recruited young university students, and this may limit generalizability to other demographic populations. The participants were well educated and informed about placebos, which was reflected in the sophisticated answers of the participants (e.g., only one participant was not aware of the term "placebo"). A recent survey study also found that a higher education is related to more placebo knowledge [68]. Second, the present qualitative study examined healthy participants with presumably lower motivation for pain relief than patients. Likewise, the study did not investigate attitudes of participants toward practitioners who offer OLPs, or about possible negative effects, such as feelings of stigmatization. Some patient populations may be highly resistant to being offered OLPs or offended by the idea. Third, although we did set out to compare participants' views of OLPs with the conventional OLP disclosures, we were not able to make conclusions about the fourth discussion point (i.e., *taking the pills faithfully is critical*) since the topic of adherence was not applicable in our experimental RCT. Fourth, the researcher conducting all interviews was not blind to the group allocation, probably leading to a bias in the framing of the questions. Therefore, we decided that the two main coders were neither researchers who delivered the treatment nor those who conducted the interviews. Despite these limitations, this qualitative study offers a novel perspective on participants' views about openly prescribed placebos and contributes to the discussion regarding the use and framing of the OLP treatment rationale. The qualitative approach that we chose to reflect on the conventional OLP disclosures

For peer review only

is one possibility. Other ways to further make suggestions for the adaption of the rationale might be to integrate newest evidence-based findings of OLP trials or to focus on ethical considerations.

Conclusion

Participants of our experimental OLP trial spontaneously expressed a wide array of placebo definitions, examples, and underlying mechanisms. In their descriptions, they went beyond theoretical speculations and provided a variety of personal experiences. Given the big variety of conceptualizations, but upholding standards of evidence, clinical practice might profit from tailoring the provided information to the specific needs of individual patients, which is in line with the recommendations formulated by a placebo expert group [74]. We were able to link participants' quotations to the conventional OLP rationale. Overall, we propose that the OLP rationale would gain from further adaptions and should be revisited. We suggest that participants of OLP trials should be informed of *multiple* placebo mechanisms that exist, including but not limited to classical conditioning. Conceivably, this might further increase the plausibility of the rationale and will also be in line with newest evidence. Furthermore, and in order to set realistic expectations, we propose that participants should be motivated to "just give it a try". This is in

For peer review only

contrast to the current strategy of the rationale to decrease possible skepticism. Considering that this qualitative analysis stems from an experimental trial, clinical implications should be outlined with caution: Our findings provide preliminary indications that the OLP approach might only be suitable for a subgroup of patients who are not too skeptical about the efficacy of the OLP therapy and who are ready "to just give it a try" – maybe also because they feel that OLPs are their last treatment option. Further research should move beyond conceptualizations of placebos, and opinions about their effectiveness, to attitudes about the acceptability of being offered OLPs, especially for patients living with a variety of placebo-responsive symptoms. Such studies should explore perceptions about the competence of clinicians who offer OLPs. We also recommend that future research solicits the views of patients drawn from different demographic populations.

Ethical Approval Statement

The study was approved by the Local Ethics Committee of the Canton Basel, Switzerland (EKNZ 2015-246).

For peer review only

References

1 Enck P, Bingel U, Schedlowski M, *et al.* The placebo response in medicine: minimize, maximize or personalize? *Nat Rev Drug Discov* 2013;**12**:191–204. doi:10.1038/nrd3923
2 Locher C, Gaab J, Blease C. When a Placebo Is Not a Placebo: Problems and Solutions to the Gold Standard in Psychotherapy Research. *Frontiers in Psychology* 2018;**9**:2317. doi:10.3389/fpsyg.2018.02317
3 Finniss DG, Kaptchuk TJ, Miller F, *et al.* Biological, clinical, and ethical advances of placebo effects. *Lancet* 2010;**375**:686–95. doi:10.1016/S0140-6736(09)61706-2
4 Foddy B. A Duty to Deceive: Placebos in Clinical Practice. *Am J Bioeth* 2009;**9**:4–12. doi:10.1080/15265160903318350
5 O'Neill O. Paternalism and partial autonomy. *J Med Ethics* 1984;**10**:173–8. doi:10.1136/jme.10.4.173
6 Blease CR. The principle of parity: the 'placebo effect' and physician communication. *J Med Ethics* 2012;**38**:199–203. doi:10.1136/medethics-2011-100177
7 Bok S. The Ethics of Giving Placebos. *Sci Am* 1974;**231**:17–23. doi:10.1038/scientificamerican1174-17
8 Gaab J, Blease C, Locher C, *et al.* Go open: A plea for transparency in psychotherapy. *Psychol Conscious (Wash D C)* 2016;**3**:175–98. doi:doi.org/10.1037/cns0000063
9 Schöne-Seifert B. Placebos and placeboids in clinical practice: conceptual and ethical considerations. *Nervenarzt* 2020;**91**:684–90. doi:10.1007/s00115-020-00943-8
10 Blease CR, Bernstein MH, Locher C. Open-label placebo clinical trials: is it the rationale, the interaction or the pill? *BMJ Evid Based Med* 2019;**25**:159–65. doi:10.1136/bmjebm-2019-111209
11 Carvalho C, Caetano JM, Cunha L, *et al.* Open-label placebo treatment in chronic low back pain: A randomized controlled trial. *Pain* 2016;**157**:2766–72. doi:10.1097/j.pain.0000000000000700
12 Kleine-Borgmann J, Schmidt K, Hellmann A, *et al.* Effects of open-label placebo on pain, functional disability, and spine mobility in patients with chronic back pain: a randomized controlled trial. *Pain* 2019;**160**:2891–7. doi:10.1097/j.pain.0000000000001683
13 Kaptchuk TJ, Friedlander E, Kelley JM, *et al.* Placebos without deception: a randomized controlled trial in irritable bowel syndrome. *PLoS One* 2010;**5**:e15591. doi:https://doi.org/10.1371/journal.pone.0015591
14 Kube T, Hofmann VE, Glombiewski JA, *et al.* Providing open-label placebos remotely—A randomized controlled trial in allergic rhinitis. *PLOS ONE* 2021;**16**:e0248367. doi:10.1371/journal.pone.0248367
15 Schaefer M, Harke R, Denke C. Open-Label Placebos Improve Symptoms in Allergic Rhinitis: A Randomized Controlled Trial. *Psychother Psychosom* 2016;**85**:373–4. doi:10.1159/000447242
16 Schaefer M, Sahin T, Berstecher B. Why do open-label placebos work? A randomized controlled trial of an open-label placebo induction with and without extended information about the placebo effect in allergic rhinitis. *PLoS One* 2018;**13**:e0192758. doi:10.1371/journal.pone.0192758
17 Sandler AD, Glesne CE, Bodfish JW. Conditioned placebo dose reduction: A new treatment in attention-deficit hyperactivity disorder? *J Dev Behav Pediatr* 2010;**31**:369–75. doi:10.1097/DBP.0b013e3181e121ed

For peer review only

18 Sandler AD, Bodfish JW. Open-label use of placebos in the treatment of ADHD: A pilot study. *Child Care Hlth Dev* 2008;**34**:104–10. doi:10.1111/j.1365-2214.2007.00797.x
19 Hoenemeyer TW, Kaptchuk TJ, Mehta TS, *et al.* Open-Label Placebo Treatment for Cancer-Related Fatigue: A Randomized-Controlled Clinical Trial. *Sci Rep* 2018;**8**:2784. doi:10.1038/s41598-018-20993-y
20 Zhou ES, Hall KT, Michaud AL, *et al.* Open-label placebo reduces fatigue in cancer survivors: a randomized trial. *Support Care Cancer* 2018;**27**:2179–87. doi:10.1007/s00520-018-4477-6
21 Charlesworth JEG, Petkovic G, Kelley JM, *et al.* Effects of placebos without deception compared with no treatment: A systematic review and meta-analysis. *J Evid Based Med* 2017;**10**:97–107. doi:10.1111/jebm.12251
22 von Wernsdorff M, Loef M, Tuschen-Caffier B, *et al.* Effects of open-label placebos in clinical trials: a systematic review and meta-analysis. *Sci Rep* 2021;**11**:3855. doi:10.1038/s41598-021-83148-6
23 Pan Y, Meister R, Löwe B, *et al.* Open-label placebos for menopausal hot flushes: a randomized controlled trial. *Sci Rep* 2020;**10**:20090. doi:10.1038/s41598-020-77255-z
24 Kleine-Borgmann J, Schmidt K, Billinger M, *et al.* Effects of open-label placebos on test performance and psychological well-being in healthy medical students: a randomized controlled trial. *Sci Rep* 2021;**11**:1–11. doi:10.1038/s41598-021-81502-2
25 Schaefer M, Denke C, Harke R, *et al.* Open-label placebos reduce test anxiety and improve self-management skills: A randomized-controlled trial. *Sci Rep* 2019;**9**:13317. doi:10.1038/s41598-019-49466-6
26 El Brihi J, Horne R, Faasse K. Prescribing Placebos: An Experimental Examination of the Role of Dose, Expectancies, and Adherence in Open-Label Placebo Effects. *Ann Behav Med* 2019;**53**:16–28. doi:10.1093/abm/kay011
27 Mathur A, Jarrett P, Broadbent E, *et al.* Open-label placebos for wound healing: a randomized controlled trial. *Ann Behav Med* 2018;**52**:902–8. doi:10.1093/abm/kax057
28 Disley N, Kola-Palmer S, Retzler C. A comparison of open-label and deceptive placebo analgesia in a healthy sample. *J Psychosom Res* 2021;**140**:110298. doi:10.1016/j.jpsychores.2020.110298
29 Leibowitz KA, Hardebeck EJ, Goyer JP, *et al.* The role of patient beliefs in open-label placebo effects. *Health Psychol* 2019;**38**:613–22. doi:10.1037/hea0000751
30 Locher C, Frey Nascimento A, Kirsch I, *et al.* Is the rationale more important than deception? A randomized controlled trial of open-label placebo analgesia. *Pain* 2017;**158**:2320–8. doi:10.1097/j.pain.0000000000001012
31 Meeuwis SH, van Middendorp H, van Laarhoven AIM, *et al.* Effects of Open- and Closed-Label Nocebo and Placebo Suggestions on Itch and Itch Expectations. *Front Psychol* 2019;:436. doi:10.3389/fpsyt.2019.00436
32 Kaptchuk TJ. Open-Label Placebo: Reflections on a Research Agenda. *Perspect Biol Med* 2018;**61**:311–34. doi:10.1353/pbm.2018.0045
33 Benedetti F, Pollo A, Lopiano L, *et al.* Conscious Expectation and Unconscious Conditioning in Analgesic, Motor, and Hormonal Placebo/Nocebo Responses. *J Neurosci* 2003;**23**:4315–23. doi:10.1523/JNEUROSCI.23-10-04315.2003
34 Schedlowski M, Enck P, Rief W, *et al.* Neuro-Bio-Behavioral Mechanisms of Placebo and Nocebo Responses: Implications for Clinical Trials and Clinical Practice. *Pharmacol Rev* 2015;**67**:697–730. doi:10.1124/pr.114.009423

For peer review only

35 Kaptchuk TJ. Placebo studies and ritual theory: a comparative analysis of Navajo, acupuncture and biomedical healing. *Philos Trans R Soc Lond B Biol Sci* 2011;**366**:1849–58. doi:10.1098/rstb.2010.0385
36 Thompson JJ, Ritenbaugh C, Nichter M. Reconsidering the Placebo Response from a Broad Anthropological Perspective. *Cult Med Psychiatry* 2009;**33**:112–52. doi:10.1007/s11013-008-9122-2
37 Blease CR, Kelley JM, Trachsel M. Informed Consent in Psychotherapy: Implications of Evidence-Based Practice. *J Contemp Psychother* 2018;**48**:69–78. doi:10.1007/s10879-017-9372-9
38 Kaptchuk TJ, Shaw J, Kerr CE, *et al.* "Maybe I Made Up the Whole Thing": Placebos and Patients' Experiences in a Randomized Controlled Trial. *Cult Med Psychiatry* 2009;**33**:382–411. doi:10.1007/s11013-009-9141-7
39 Paterson C, Zheng Z, Xue C, *et al.* "Playing Their Parts": The Experiences of Participants in a Randomized Sham-Controlled Acupuncture Trial. *J Altern Complement Med* 2008;**14**:199–208. doi:10.1089/acm.2007.0682
40 Bernstein MH, Magill M, Weiss A-P, *et al.* Are conditioned open placebos feasible as an adjunctive treatment to opioids? Results from a single-group dose-extender pilot study with acute pain patients. *Psychother Psychosom* 2019;**88**:380–2. doi:10.1159/000503038
41 VERBI Software. (2019). MAXQDA 2020 [computer software]. Berlin, Germany: VERBI Software. MAXQDA. 2019.maxqda.com (accessed 19 Dec 2020).
42 Fereday J, Muir-Cochrane E. Demonstrating Rigor Using Thematic Analysis: A Hybrid Approach of Inductive and Deductive Coding and Theme Development. *Int J Qual Methods* 2006;**5**:80–92. doi:10.1177/160940690600500107
43 Hsieh H-F, Shannon SE. Three Approaches to Qualitative Content Analysis. *Qual Health Res* 2005;**15**:1277–88. doi:10.1177/1049732305276687
44 Oakley A, Strange V, Bonell C, *et al.* Process evaluation in randomised controlled trials of complex interventions. *BMJ* 2006;**332**:413–6. doi:10.1136/bmj.332.7538.413
45 O'Cathain A. *A Practical Guide to Using Qualitative Research with Randomized Controlled Trials*. 1st ed. Oxford University Press 2018. https://oxfordmedicine.com/view/10.1093/med/9780198802082.001.0001/med-9780198802082
46 Elo S, Kyngäs H. The qualitative content analysis process. *J Adv Nurs* 2008;**62**:107–15. doi:https://doi.org/10.1111/j.1365-2648.2007.04569.x
47 Braun V, Clarke V. Using thematic analysis in psychology. *Qual Res Psychol* 2006;**3**:77–101. doi:10.1191/1478088706qp063oa
48 Locher C, Frey Nascimento A, Kossowsky J, *et al.* Open-label placebo response – Does optimism matter? A secondary-analysis of a randomized controlled trial. *J Psychosom Res* 2019;**116**:25–30. doi:10.1016/j.jpsychores.2018.11.009
49 Annoni M, Blease C. A Critical (and Cautiously Optimistic) Appraisal of Moerman's "Meaning Response." *Perspect Biol Med* 2018;**61**:379–87. doi:10.1353/pbm.2018.0050
50 Brody H. The placebo response: Recent research and implications for family medicine. *J Fam Pract* 2000;**49**:649–54.
51 Howick J. The relativity of 'placebos': defending a modified version of Grünbaum's definition. *Synthese* 2017;**194**:1363–96. doi:10.1007/s11229-015-1001-0
52 Kirsch I, Wampold B, Kelley JM. Controlling for the placebo effect in psychotherapy: Noble quest or tilting at windmills? *Psychol Conscious (Wash D C)* 2016;**3**:121–31. doi:10.1037/cns0000065

For peer review only - http://bmjopen.bmj.com/site/about/guidelines.xhtml

53 Miller FG, Colloca L. The Legitimacy of Placebo Treatments in Clinical Practice: Evidence and Ethics. *Am J Bioeth* 2009;**9**:39–47. doi:10.1080/15265160903316263
54 Crum AJ, Langer EJ. Mind-Set Matters: Exercise and the Placebo Effect. *Psychol Sci* 2007;**18**:165–71. doi:10.1111/j.1467-9280.2007.01867.x
55 Goldberg SB, Tucker RP, Greene PA, *et al.* Mindfulness-based interventions for psychiatric disorders: A systematic review and meta-analysis. *Clin Psychol Rev* 2018;**59**:52–60. doi:10.1016/j.cpr.2017.10.011
56 Birkhäuer J, Gaab J, Kossowsky J, *et al.* Trust in the health care professional and health outcome: A meta-analysis. *PLOS ONE* 2017;**12**:e0170988. doi:10.1371/journal.pone.0170988
57 Gaab J, Kossowsky J, Ehlert U, *et al.* Effects and Components of Placebos with a Psychological Treatment Rationale–Three Randomized-Controlled Studies. *Sci Rep* 2019;**9**:1421. doi:10.1038/s41598-018-37945-1
58 Kaptchuk TJ, Kelley JM, Conboy LA, *et al.* Components of placebo effect: randomised controlled trial in patients with irritable bowel syndrome. *BMJ* 2008;**336**:999–1003. doi:10.1136/bmj.39524.439618.25
59 Kelley JM, Kraft-Todd G, Schapira L, *et al.* The influence of the patient-clinician relationship on healthcare outcomes: A systematic review and meta-analysis of randomized controlled trials. *PLoS One* 2014;**9**:e94207. doi:10.1371/journal.pone.0094207
60 Locher C, Meier S, Gaab J. Psychotherapy: A World of Meanings. *Front Psychol* 2019;:460. doi:10.3389/fpsyg.2019.00460
61 Lembo A, Kelley JM, Nee J, *et al.* Open-label placebo vs double-blind placebo for irritable bowel syndrome: a randomized clinical trial. *Pain* 2021;**Articles in Press**. doi:10.1097/j.pain.0000000000002234
62 Kirsch I. Response expectancy as a determinant of experience and behavior. *Am Psychol* 1985;**40**:1189–202. doi:10.1037/0003-066X.40.11.1189
63 Kirsch I. Conditioning, Expectancy, and the Placebo Effect: Comment on Stewart-Williams and Podd (2004). *Psychol Bull* 2004;**130**:341–3. doi:10.1037/0033-2909.130.2.341
64 Petrie KJ, Rief W. Psychobiological Mechanisms of Placebo and Nocebo Effects: Pathways to Improve Treatments and Reduce Side Effects. *Annu Rev Psychol* 2019;**70**:599–625. doi:10.1146/annurev-psych-010418-102907
65 Wager TD, Atlas LY. The neuroscience of placebo effects: connecting context, learning and health. *Nat Rev Neurosci* 2015;**16**:403–18. doi:10.1038/nrn3976
66 Di Blasi Z, Crawford F, Bradley C, *et al.* Reactions to treatment debriefing among the participants of a placebo controlled trial. *BMC Health Serv Res* 2005;**5**:30. doi:10.1186/1472-6963-5-30
67 Stone DA, Kerr CE, Jacobson E, *et al.* Patient expectations in placebo-controlled randomized clinical trials. *J Eval Clin Pract* 2005;**11**:77–84. doi:10.1111/j.1365-2753.2004.00512.x
68 Smits RM, Veldhuijzen DS, Hartman TO, *et al.* Explaining placebo effects in an online survey study: Does 'Pavlov' ring a bell? *PLoS One* 2021;**16**:e0247103. doi:10.1371/journal.pone.0247103
69 Conboy L, Quilty MT, Kerr C, *et al.* A Qualitative Analysis of Adolescents' Experiences of Active and Sham Japanese-Style Acupuncture Protocols Administered in a Clinical Trial. *J Altern Complement Med* 2008;**14**:699–705. doi:10.1089/acm.2007.0718
70 Franklin J. Pascal's wager and the origins of decision theory: decision-making by real decision-makers. *Phil Archive* 2018;:27–44.

For peer review only

71 Ratnapalan M, Coghlan B, Tan M, *et al.* Placebos in primary care? a nominal group study explicating UK GP and patient views of six theoretically plausible models of placebo practice. *BMJ Open* 2020;**10**:e032524. doi:10.1136/bmjopen-2019-032524

72 Thielke SM, Turner JA, Shortreed SM, *et al.* Do Patient-perceived Pros and Cons of Opioids Predict Sustained Higher-Dose Use? *Clin J Pain* 2014;**30**:93–101. doi:10.1097/AJP.0b013e31828e361b

73 Geers AL, Wellman JA, Fowler SL, *et al.* Dispositional Optimism Predicts Placebo Analgesia. *J Pain* 2010;**11**:1165–71. doi:10.1016/j.jpain.2010.02.014

74 Evers AWM, Colloca L, Blease C, *et al.* What Should Clinicians Tell Patients about Placebo and Nocebo Effects? Practical Considerations Based on Expert Consensus. *Psychother Psychosom* 2021;**90**:49–56. doi:10.1159/000510738

For peer review only

Table 1
Sociodemographic characteristics of study participants

Group	N	Age (SD)	N (%) female	Family status	Highest educational level N	Employment level N	Credibility of the intervention N
OPR-	10	25.3 (6.63)	6 (60%)	Single: 10	Primary school: 0<br>Secondary school: 1<br>High school: 5<br>University: 4	Full time: 0<br>Part time: 3<br>None or student: 7	credible: 8<br>somewhat credible: 2<br>not credible: 0
DP	10	30.9 (14.65)	6 (60%)	Single: 9<br>Divorced: 1	Primary school: 1<br>Secondary school: 0<br>High school: 5<br>University: 4	Full time: 0<br>Part time: 5<br>None or student: 5	credible: 6<br>somewhat credible: 3<br>not credible: 1
OPR+	10	25.4 (4.86)	7 (70%)	Single: 10	Primary school: 0<br>Secondary school: 1<br>High school: 5<br>University: 4	Full time: 1<br>Part time: 5<br>None or student: 4	credible: 9<br>somewhat credible: 1<br>not credible: 0

Note. DP, deceptive placebo; OPR-, open-label placebo without rationale; OPR+, open-label placebo with rationale.

Table 2
Superordinate categories with corresponding main categories

Superordinate Categories	Main Categories
1. The placebo concept.	1.1. Descriptive
	1.2. Effectiveness
	1.3 Specific examples
2. Open-label placebo rationale.	2.1 First discussion point: "Placebos are powerful"
	2.2 Second discussion point: "The body automatically responds to placebos like Pavlov's dog"
	2.3 Third discussion point: "A positive attitude helps but is not necessary"
3. Experiences of taking part in an open-label placebo trial.	3.1. Reaction to (open-label) placebo
	3.2. Efficacy and effectiveness during the experiment

For peer review only

For peer review only

eAppendix 1. Provided rationales

Translated from German into English

Deceptive Placebo (DP) group:

Now, before you take the pain measurements for the second time, I would like to explain you the treatment approach. You will now receive a cream for pain relief. It is a generic product with the active ingredient lidocaine, which is also contained in creams like “Stilex”. The “antidolor cream” is used to prevent and treat painful and itchy skin conditions such as first-degree burns, minor non-extensive sunburn and insect bites. The effect of lidocaine has been proven in multiple high-quality studies.

OPR- group:

Now, before you take the pain measurements for the second time, you will receive a placebo cream. This means that you will be given a cream that does not contain any medicinal active ingredient and that is therefore inactive.

OPR+ group:

Now, before we take the pain measurements for the second time, I would like to explain you the treatment approach. You will now receive a placebo cream. Have you ever heard of placebo? Placebo is something that does not contain an active ingredient and is inactive, but still works. Now I will give you an overview of newest research findings:

First discussion point:

It has been shown that placebos can work just as well as real drugs, especially in the fields of pain, Parkinson's disease, depression, migraine and asthma. For example, in the field of pain: Research indicates that the placebo effect is involved in 50% of the effect of pain pills and creams [Mention two studies: Colloca et al., 2013; Benedetti et al., 2001].

Conclusively, placebos lead to measurable biological reactions in the body and are much more than just imagination. So, you could say that placebos boost the "body's own pharmacy".

But it gets even more interesting - placebos even work when you know it's a placebo. This is the approach we are taking in our experiment. For our study, we are working closely with the research group from Harvard. In a landmark study of 2010, it was shown that patients with

For peer review only

irritable bowel syndrome had a significant decrease in symptoms, even when they were openly informed that they were receiving a placebo [Kaptchuk et al., 2010].

Second discussion point:

Now, I would like to tell you about *how* placebos work. One explanation for these processes is classical conditioning. Maybe you heard about the experiment of Pavlov and his dog. The dog is given food several times with a bell ringing. Later, the pure bell ringing leads to salivation, even if the dog does not receive food. Comparable is the physiological reaction of our body to placebos: From an early age we learn that the application of a cream and the effect are associated. Finally, the mere application of the cream results in pain relief. Similar to the Pavlov's dog, we therefore perform a ritual.

Third discussion point:

The discussed treatment approach may sound a bit unusual at first sight. Another plus point of placebos is that a positive attitude helps but is not necessary.

For peer review only

eAppendix 2. Topic guide of the qualitative interview

1. Discourse

In general:

In your own words: what do you imagine a placebo to be?	
How do you think placebos work?	
What could be the cause/reason?	

Related to the experiment:

How did the experiment influence your understanding of placebos?	

2. Project

In general:

To what extent do placebos occur in your everyday life? (Active use of the placebo effect - e.g., in the form of home remedies)	
How significant are these effects and implications of a placebo for you?	

Related to the experiment:

Now if you had pain (e.g., heat pain, burning sensation) - would you use a cream described as "placebo"?	
If yes: Would you have taken this cream before experiencing this experiment?	

3. Selfhood

In general:

Do you think you respond particularly well/badly to placebos? Why?	
How much control do you think you have over the effect of a placebo?	

Related to the experiment:

What do you think - did the placebo cream have an effect on pain perception in this study? If so, what was the effect?	

4. Embodiment

In general:

For peer review only

Do you believe that a placebo can have an "effective" physical effect or is it merely an "imaginary" effect, i.e., a deception (of the senses)?	

Related to the experiment:

What was it like for you? Please describe the physical sensations of using the placebo cream.	

5. Sociality

In general:

To which people in your social environment would you recommend placebos? Why?	

Related to the experiment:

Will you tell people at home (with friends or relatives) about your experience with placebos that you had in the experiment?	
How will this make you feel towards the other person?	

6. Spatiality

In general:

Can you describe to me an environment or place where you feel, a placebo would work particularly well?	

Related to the experiment:

What impact did it have on you that you received the placebo in the context of an experiment and not in the "real world"?	

7. Temporality

In general:

How long do you think placebos last?	
When you think back - what significant memories with placebos come to mind? (e.g., comforting songs in childhood, remedies for a cold)	

Related to the experiment:

A glimpse into the future: Do you take anything away from the experiment and from this conversation with respect to placebos?	

For peer review only

eTable 1. Superordinate categories, main categories and categories

Superordinate categories, main categories and categories

Superordinate Categories	Main Categories	Categories	Frequencies		
			OPR-	OPR+	DP
1. The placebo concept.	1.1. Descriptive	Deception	4	6	10
		Study design component	1	0	1
		Inertness	7	9	6
		Substitute	2	0	0
	1.2. Effectiveness	Regularity of intake is necessary for effectiveness	2	1	2
		Sham effect	0	0	2
		Effect on body	9	3	7
		Psychological effect	3	1	1
		Paradox: Effect without active ingredient	6	5	1
		Effect-free	5	0	1
	1.3 Specific examples	Autosuggestion	1	1	1
		Spirituality	1	1	1
		Drug	0	4	2
		Interpersonal component	2	2	5
		Esotericism	1	0	1
		Deception	0	0	1
		Advertisement	0	0	1
		Complementary medicine	8	8	9
		Action	0	4	6
		Activity	2	3	6
		Food supplements	0	2	2
		Mindfulness	1	0	0
		Food products	9	8	11
2. Open-label placebo rationale.	2.1 First discussion point: “Placebos are powerful”	Less efficacy due to lack of deception	4	2	5
		OLP administration mitigates expectancy/belief	4	2	5
		OLPs work	0	1	0
		Surprise about OLPs effectiveness	0	2	0
		Effect of deceptive placebos lasts longer in contrast to OLPs	0	2	0
		Effect due to deceptive placebos, less effect due to OLPs	0	2	0
	2.2 Second discussion point: “The body automatically responds to placebos like Pavlov’s dog”	Relationship	9	6	7
		Imagination	0	2	2
		Psychological factors	11	11	7
		Misattribution	1	0	0
		Mind-body-interaction	22	17	13
		Conditioning	4	11	2
		Ritual/action	3	5	1
		Openness/personality/attitudes	37	24	26
		Expectancy/belief/hope	28	27	30
	2.3 Third discussion	Depending on the experience	2	6	1
		If it helps and does no harm	0	0	1

For peer review only

	point: "A positive attitude helps but is not necessary"	If it's the only option	2	3	2
		It's worth a try	3	2	0
		Worth to see what happens	0	2	0
		As long as one believes that it helps	2	0	3
		Depending on the person	2	0	1
		Depending on the situation	0	1	0
		Depending on symptoms / disorder	4	4	3
3. Experiences of taking part in an open-label placebo trial.	3.1. Reaction to (open-label) placebo	When administered openly, skepticism arises as to whether an ingredient is not in the pill after all	2	1	0
		OLPs do not elicit wellbeing	0	1	0
		OLPs as something novel	1	6	0
		OLPs are incomprehensible/strange/crazy	5	1	1
		Openness towards OLPs	0	1	0
		Open administration is less popular	0	1	0
		OLP rationale is essential	0	3	0
		Placebo as something unknown	0	0	2
	3.2. Efficacy and effectiveness during the experiment	No efficacy due to the lack of deception	0	1	0
		Efficacy unlikely	1	3	0
		Efficacy only assumed posthoc	0	0	2
		Efficacy possible	4	1	4
		Efficacy unclear	1	3	0
		No objective efficacy (measures), but subjective efficacy (own sensation)	0	0	1
		Efficacy likely	1	0	4
		Positive experience due to cooling/experimental factors	4	3	4
		Positive experience on a psychological level	1	2	0
		No experienced efficacy in the experiment	9	9	5

Note. DP = deceptive placebo; OPR- = open-label placebo without rationale; OPR+ = open-label placebo with rationale.

For peer review only

Standards for Reporting Qualitative Research (SRQR)*

http://www.equator-network.org/reporting-guidelines/srqr/

Page/line no(s).

Title and abstract

**Title** - Concise description of the nature and topic of the study Identifying the study as qualitative or indicating the approach (e.g., ethnography, grounded theory) or data collection methods (e.g., interview, focus group) is recommended	p. 1 / line 1
**Abstract** - Summary of key elements of the study using the abstract format of the intended publication; typically includes background, purpose, methods, results, and conclusions	p. 2 / lines 26-44

Introduction

**Problem formulation** - Description and significance of the problem/phenomenon studied; review of relevant theory and empirical work; problem statement	p. 4-5 / lines 71-117
**Purpose or research question** - Purpose of the study and specific objectives or questions	p. 6 / lines 118-125

Methods

**Qualitative approach and research paradigm** - Qualitative approach (e.g., ethnography, grounded theory, case study, phenomenology, narrative research) and guiding theory if appropriate; identifying the research paradigm (e.g., postpositivist, constructivist/ interpretivist) is also recommended; rationale**	p. 7-8 / lines 157-162
**Researcher characteristics and reflexivity** - Researchers' characteristics that may influence the research, including personal attributes, qualifications/experience, relationship with participants, assumptions, and/or presuppositions; potential or actual interaction between researchers' characteristics and the research questions, approach, methods, results, and/or transferability	p. 7 / lines 153-156
**Context** - Setting/site and salient contextual factors; rationale**	
**Sampling strategy** - How and why research participants, documents, or events were selected; criteria for deciding when no further sampling was necessary (e.g., sampling saturation); rationale**	p. 6-7/ lines 133-139
**Ethical issues pertaining to human subjects** - Documentation of approval by an appropriate ethics review board and participant consent, or explanation for lack thereof; other confidentiality and data security issues	p. 6 / lines 131-132
**Data collection methods** - Types of data collected; details of data collection procedures including (as appropriate) start and stop dates of data collection and analysis, iterative process, triangulation of sources/methods, and modification of procedures in response to evolving study findings; rationale**	p. 7 / lines 142-145

For peer review only - http://bmjopen.bmj.com/site/about/guidelines.xhtml

**Data collection instruments and technologies** - Description of instruments (e.g., interview guides, questionnaires) and devices (e.g., audio recorders) used for data collection; if/how the instrument(s) changed over the course of the study	p. 7 / lines 145-149
**Units of study** - Number and relevant characteristics of participants, documents, or events included in the study; level of participation (could be reported in results)	p. 8-9 / lines 172-189
**Data processing** - Methods for processing data prior to and during analysis, including transcription, data entry, data management and security, verification of data integrity, data coding, and anonymization/de-identification of excerpts	p. 7 / lines 150-152
**Data analysis** - Process by which inferences, themes, etc., were identified and developed, including the researchers involved in data analysis; usually references a specific paradigm or approach; rationale**	p. 8 / lines 163-171
**Techniques to enhance trustworthiness** - Techniques to enhance trustworthiness and credibility of data analysis (e.g., member checking, audit trail, triangulation); rationale**	p. 8 / line 171

Results/findings

**Synthesis and interpretation** - Main findings (e.g., interpretations, inferences, and themes); might include development of a theory or model, or integration with prior research or theory	p. 9-18 / lines 191-391
**Links to empirical data** - Evidence (e.g., quotes, field notes, text excerpts, photographs) to substantiate analytic findings	p. 18 / lines 402-404

Discussion

**Integration with prior work, implications, transferability, and contribution(s) to the field -** Short summary of main findings; explanation of how findings and conclusions connect to, support, elaborate on, or challenge conclusions of earlier scholarship; discussion of scope of application/generalizability; identification of unique contribution(s) to scholarship in a discipline or field	p. 18-23 / lines 392-508
**Limitations** - Trustworthiness and limitations of findings	p. 23 / lines 509-525

Other

**Conflicts of interest** - Potential sources of influence or perceived influence on study conduct and conclusions; how these were managed	p. 3 / line 65
**Funding** - Sources of funding and other support; role of funders in data collection, interpretation, and reporting	p. 3 / lines 63-64

*The authors created the SRQR by searching the literature to identify guidelines, reporting standards, and critical appraisal criteria for qualitative research; reviewing the reference lists of retrieved sources; and contacting experts to gain feedback. The SRQR aims to improve the transparency of all aspects of qualitative research by providing clear standards for reporting qualitative research.

For peer review only - http://bmjopen.bmj.com/site/about/guidelines.xhtml

**The rationale should briefly discuss the justification for choosing that theory, approach, method, or technique rather than other options available, the assumptions and limitations implicit in those choices, and how those choices influence study conclusions and transferability. As appropriate, the rationale for several items might be discussed together.

Reference:

O'Brien BC, Harris IB, Beckman TJ, Reed DA, Cook DA. **Standards for reporting qualitative research: a synthesis of recommendations.** *Academic Medicine*, Vol. 89, No. 9 / Sept 2014 DOI: 10.1097/ACM.0000000000000388

For peer review only
